# Kinetically-Defined Component Actions in Gene Repression

**DOI:** 10.1371/journal.pcbi.1004122

**Published:** 2015-03-27

**Authors:** Carson C. Chow, Kelsey K. Finn, Geoffery B. Storchan, Xinping Lu, Xiaoyan Sheng, S. Stoney Simons

**Affiliations:** 1 Mathematical Biology Section, NIDDK/LBM, National Institutes of Health, Bethesda, Maryland, United States of America; 2 Steroid Hormones Section, NIDDK/LERB, National Institutes of Health, Bethesda, Maryland, United States of America; University of Illinois at Urbana-Champaign, UNITED STATES

## Abstract

Gene repression by transcription factors, and glucocorticoid receptors (GR) in particular, is a critical, but poorly understood, physiological response. Among the many unresolved questions is the difference between GR regulated induction and repression, and whether transcription cofactor action is the same in both. Because activity classifications based on changes in gene product level are mechanistically uninformative, we present a theory for gene repression in which the mechanisms of factor action are defined kinetically and are consistent for both gene repression and induction. The theory is generally applicable and amenable to predictions if the dose-response curve for gene repression is non-cooperative with a unit Hill coefficient, which is observed for GR-regulated repression of AP1LUC reporter induction by phorbol myristate acetate. The theory predicts the mechanism of GR and cofactors, and where they act with respect to each other, based on how each cofactor alters the plots of various kinetic parameters vs. cofactor. We show that the kinetically-defined mechanism of action of each of four factors (reporter gene, p160 coactivator TIF2, and two pharmaceuticals [NU6027 and phenanthroline]) is the same in GR-regulated repression and induction. What differs is the position of GR action. This insight should simplify clinical efforts to differentially modulate factor actions in gene induction vs. gene repression.

## Author Summary

While the initial steps in steroid-regulated gene induction and repression are known to be identical, the same cannot be said of cofactors that modulate steroid-regulated gene activity. We describe the conditions under which a theoretical model for gene repression reveals the kinetically-defined mechanism and relative position of cofactor action. This theory has been validated by experimental results with glucocorticoid receptors. The mode and position of action of four factors is qualitatively identical in gene repression to that previously found in gene induction. What changes is the position of GR action. Therefore, we predict that the same kinetically-defined mechanism usually will be utilized by cofactors in both induction and repression pathways. This insight and simplification should facilitate clinical efforts to maximize desired outcomes in gene induction or repression.

## Introduction

The initial steps by which steroid receptors induce or repress target gene transcription are the same. After steroid binding to the intracellular receptor, the resulting complex is activated/transformed to a form with increased affinity for DNA [1] and is concentrated in the nucleus, where it is recruited to DNA sequences that are usually near the regulated genes. Cofactors and comodulators assist or impede the transcriptional activity of DNA-associated steroid receptors [2,3]. Beyond this, it is currently not possible to predict the transcriptional outcome for any specific combination of gene, receptor, and cofactor/comodulator. In most cells, a given steroid-bound receptor will induce one set of genes while repressing another set under otherwise identical conditions. For certain genes, the same receptor-steroid complex activates transcription in one cell line while repressing it in another cell line [4]. Similarly, selected cofactors increase the activity of one steroid receptor while reducing the activity of another receptor [5]. In some cases, different cofactors cause the same gene to be induced or repressed [6,7]. In other cases, the same cofactor may interact with the same steroid receptor to augment the induction of one gene but increase the repression of another gene [6–10]. Thus no relationship between induction vs. repression and presence of particular promoter/enhancer bound factors and cofactors has yet emerged [11]. The DNA sequence to which the receptor is recruited, either by direct DNA binding or by tethering to another DNA-bound molecule, can often indicate the resultant activity of induction or repression respectively [11–13]. Even this categorization, though, is not precise as repression can occur from GR binding directly to DNA [14,15] and the outcomes can depend upon whether the cofactor binds to DNA-bound glucocorticoid receptor (GR) or GR is tethered to DNA-bound cofactor [16].

An unresolved question is whether the underlying mechanism of each transcriptional component is the same or changes with the direction of gene expression output (i.e., increase in induction vs. decrease in repression). The answers are vital because efforts to modify the responses with selected factor combinations, be it in isolated cells or human patients, will depend critically on whether the mechanism of each component is constant or varies with the specific mixture of factors. Current attempts to address these issues have been inhibited by insufficiently precise methods of analysis. Thus, many cofactors are classified as either coactivators or corepressors based solely upon their ability to increase or decrease respectively, the level of steroid-mediated gene expression [2,17]. Unfortunately, while such descriptions are operationally useful, they are mechanistically uninformative. It is well known from enzyme kinetics that an enzymatic inhibitor can increase the total response while an enzymatic activator can lead to decreased output [18–20].

A more precise and quantitative understanding of the mechanisms of gene transcription is required to resolve these issues. Mathematical modeling provides one solution; and, a theory has been developed recently to understand the underlying mechanisms of factor action during steroid-regulated gene induction. The theory is based on the fact that the dose-response curve for gene induction is non-cooperative with a Hill coefficient of one [20]. This shape of the dose-response curve has also been variously described as a Michaelis-Menten function, hyperbolic dose-response, first-order Hill plot, and first-order Hill dose-response. The essential feature is that it is in the mathematical family of linear-fractional functions.

**Fig 1:**
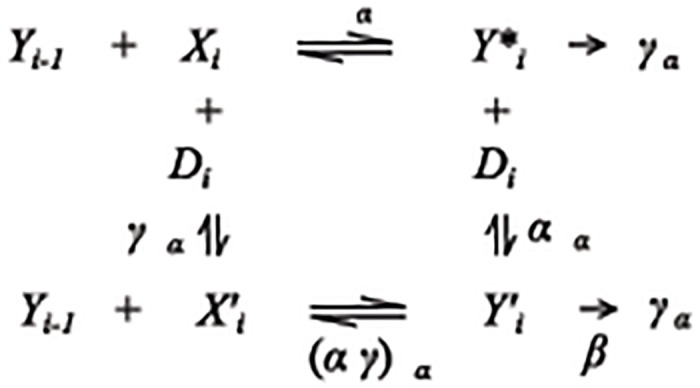
Reaction diagram for a single step in the gene induction sequence. The product from the previous step *Y*_*i-1*_ combines with the accelerator *X*_*i*_ to form a new product $Y_{i}^{*}$ with equilibrium or affinity constant *q*_*i*_. A decelerator *D*_*i*_ inhibits the reaction by binding to *X*_*i*_ to form $X_{i}^{'}$ with affinity constant ${\gamma q}_{i}^{'}$ and/or by binding to $Y_{i}^{*}$ to form $Y_{i}^{'}$. The two products $Y_{i}^{'}$ and $Y_{i}^{*}$ enter the following reaction in the combination $\left[Y_{i}\right]=\left[Y_{i}^{*}\right]+\beta \left[Y_{i}^{'}\right]$. The decelerator is called competitive when α = 0, uncompetitive when γ = 0, noncompetitive when α = γ, linear when β = 0, and partial when β>0.

The theory enables one to determine the kinetically-defined mechanism of factor action and the position of factor action in the many steps of the overall signaling cascade. This position is specified relative to both another competing factor and a steady-state analogue of a rate-limiting step called the concentration limited step (CLS) [20–27]. The concentrations of bound factors after the CLS are negligible compared to their unbound concentrations (which could be involved in other reactions as long as they are readily available). A factor can act kinetically like an enzymatic activator, which we call accelerator, or an enzymatic inhibitor, which we call decelerator, depending on how the factor participates in the reaction [23] (see Fig. 1). These classifications describe how the factor alters a specific reaction step independently of the direction of change in the observed product. In all cases investigated so far, the reporter acts as an accelerator at the CLS and thus serves as an invariant positional landmark in the otherwise poorly defined landscape of reactions in steroid-regulated gene induction [23,25–27].

Here, we extend the mathematical theory and model to GR-regulated gene repression as influenced by varying concentrations of competing cofactors. The relevant mass-action equations have been used to relate the graphs of several reaction parameters derived from the dose-response to the kinetically-defined mechanism and position of action of each of the two competing cofactors. Using the extensively characterized system of GR repression of AP1 induction in U2OS.rGR cells with a transiently transfected synthetic reporter [8,15,28,29], we show that four factors (the reporter gene, TIF2, and two small molecules [30]) have the same kinetically-defined mechanism and position of action as in GR-regulated gene induction. The difference between induction and repression is the position of GR action. This suggests that many transcriptional cofactors/comodulators are mono-functional and similarly modulate basic steps in gene expression irrespective of the directional change in gene product levels.

## Results

### Non-cooperative dose-response

**Fig 2:**
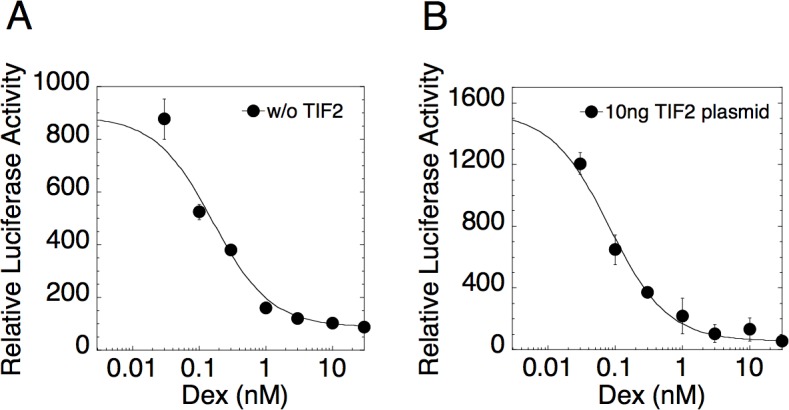
Dose-response curve for GR repression (for eight doses of Dex) is linear-fractional plot. U2OS.rGR cells were transiently transfected with 35ng of AP1LUC plasmid and (A) 0ng or (B) 10ng of TIF2 plasmid and then treated with 15ng/ml of PMA and the indicated concentrations of Dex. Luciferase activity was determined and the data plotted and fit to Equation (1) as described in Materials and Methods (error bars are ± S.D. of triplicates).

Experimentally, the dose-response of gene activity *A* in steroid-regulated repression has been found to be non-cooperative with a Hill coefficient of one (see Fig. 2): i.e.

$$A=\frac{A_{max}+A_{min}/IC_{50}[S]}{1+1/IC_{50}[S]}$$ (1)

where A_max_ is the maximal activity with no added steroid, A_min_ is the minimum value of activity with saturating steroid concentrations, and IC_50_ is the concentration of steroid for half-maximal suppression. Added cofactors can change the parameters A_max_, A_min_ and IC_50_ while preserving the shape of the dose-response (1). Our goal was to develop a theory for gene repression that explains why the dose-response has the shape given in (1) and what that implies for the actions of the added cofactors that change the parameters. We use the fact that the linear-fractional shape of the dose-response curve puts severe constraints on the possible biochemical kinetic schemes involved in gene repression, as it did for gene induction [20]. We then derive formulas that can be compared to the data to make predictions for the actions of cofactors.

Our data for the dose-response curve is based on the contributions from many copies of the induced gene in multiple cells. Hence, it is possible that the dose-response for a single gene could be different from the averaged population response. However, single-molecule imaging experiments for a gene induced by a nuclear receptor show that transcripts are produced in on-off stochastic bursts, where the bursting is well modeled as a stochastic process (i.e. random telegraph model [31,32]) and the burst probability is similar between cells. The frequency (stochastic intensity) of the stochastic bursts of individual genes follows a non-cooperative dose-response with respect to the inducing agonist [31,32]. Thus, the mean activity of a single gene follows the same non-cooperative dose-response and our theory explains the behavior of this mean.

### Theory of non-cooperative gene induction

We first present a theory for which gene induction has a non-cooperative dose-response since the theory for gene repression hinges directly on it. Gene expression involves the binding of molecules, protein, and DNA into complexes that lead to transcription. We model this as a sequence of complex building reactions, $Y_{i-1}+X_{i}\overset{q_{i}}{↔}Y_{i}$, where we call the *Y* variables products, the *X* variables accelerators, and the *q’s* are equilibrium or affinity constants. Decelerators of various types can also inhibit each of these reactions. Fig. 1 shows the general reaction scheme at each step. The accelerator *X* in Fig. 1 acts like an enzymatic activator and the decelerator *D* acts like an enzymatic inhibitor [23]. The kinetic scheme is stochastic and characterized by a probability distribution for the reactants. However, to simplify the calculations, we consider the mean field limit obeying the law of mass action and assume that the gene activity is proportional to the mean concentration of one or a set of products. The dose-response curve is the gene activity as a function of the concentration of the initial product [*Y*_0_] (i.e., agonist steroid).

The theory can be illustrated by an example of induction with three reactions (*i* = 1, 2, 3) in the absence of deceleration (which we introduce later). Suppose the dose-response is given by [*Y*_3_] as a function of [*Y*_0_]. The goal is to calculate this function and determine conditions for when it is non-cooperative with unit Hill coefficient. In steady state, the concentrations obey the equilibrium conditions

$$\left[Y_{i}\right]=q_{i}\left[X_{i}\right]\left[Y_{i-1}\right],i=1,2,3$$ (2)

and the mass conservation conditions

$$\begin{array}{c} \left[X_{1}\right]+\left[Y_{1}\right]+\left[Y_{2}\right]+\left[Y_{3}\right]=X_{1}^{T}\\ \left[X_{2}\right]+\left[Y_{2}\right]+\left[Y_{3}\right]=X_{2}^{T}\\ \left[X_{3}\right]+\left[Y_{3}\right]=X_{3}^{T} \end{array}$$

where $X_{i}^{T}$ is the total concentration of accelerator *i*. Together they form a system of 6 equations and 7 unknowns. Therefore, any one concentration can be solved in terms of any other. In general, the dose-response for this system will not have unit Hill coefficient [20]. However, a non-cooperative dose response can arise if [*Y*_*2*_],[*Y*_*3*_]<<[*Y*_*1*_] and [*Y*_*3*_]<<[*X*_*3*_] so that the mass conservation equations become

$$\begin{array}{c} \left[X_{1}\right]+\left[Y_{1}\right]=X_{1}^{T}\text{(3a)}\\ \left[X_{2}\right]+\left[Y_{2}\right]+\left[Y_{3}\right]=X_{2}^{T}\text{(3b)}\\ \left[X_{3}\right]=X_{3}^{T}\text{(3c)} \end{array}$$

This form for mass conservation can be achieved if the concentration of *X*_2_ is limited with respect to its binding affinity, i.e., *q*_*2*_[*X*_*2*_]<<1, while the other factors are not limited. This can be achieved biochemically if the products have short lifetimes, which has been observed experimentally [33–35]. We call Step (3b) the concentration-limited step (CLS) [20]. All factors following the CLS are in excess (i.e., bound concentrations are negligible), implying that reactions after the CLS are pseudo-first order. Hence, the CLS is a step where the accelerator concentration is limited with respect to its binding affinity but the accelerator(s) following it are not limited. In the Methods, we give a detailed account of the CLS for an arbitrary number of reactions.

Substituting (2) into (3a)–(3b) gives

$$\begin{array}{c} \left[X_{1}\right]+q_{1}\left[X_{1}\right]\left[Y_{0}\right]=X_{1}^{T}\\ \left[X_{2}\right]+q\left[X_{2}\right]\left[Y_{1}\right]+q_{3}X_{3}^{T}q_{2}\left[X_{2}\right]\left[Y_{1}\right]=X_{2}^{T}\\ \left[X_{3}\right]=X_{3}^{T} \end{array}$$

Each equation is bilinear in the accelerator and product concentrations. When the accelerator concentrations are substituted back into the equilibrium Equations (2), the results are linear-fractional functions between adjacent products:

$$\left[Y_{1}\right]=\frac{q_{1}X_{1}^{T}\left[Y_{0}\right]}{1+q_{1}\left[Y_{0}\right]},\left[Y_{2}\right]=\frac{q_{2}X_{2}^{T}\left[Y_{1}\right]}{1+q_{2}\left(1+q_{3}X_{3}^{T}\right)\left[Y_{1}\right]},\left[Y_{3}\right]=q_{3}X_{3}^{T}\left[Y_{2}\right]$$

Linear-fractional functions form a group under function composition ensuring that the function of any product in terms of any other product is always linear-fractional [20]. Successive substitution of these functions yields the dose-response:

$$A\equiv \left[Y_{3}\right]=\frac{q_{1}X_{1}^{T}q_{2}X_{2}^{T}q_{3}X_{3}^{T}\left[Y_{0}\right]}{1+q_{1}\left[Y_{0}\right]+q_{2}\left(1+q_{3}X_{3}^{T}\right)q_{1}X_{1}^{T}\left[Y_{0}\right]}$$

where

$$A_{max}=\frac{q_{1}X_{1}^{T}q_{2}X_{2}^{T}q_{3}X_{3}^{T}}{q_{1}+q_{2}\left(1+q_{3}X_{3}^{T}\right)q_{1}X_{1}^{T}},EC_{50}=\frac{1}{q_{1}+q_{2}\left(1+q_{3}X_{3}^{T}\right)q_{1}X_{1}^{T}}$$

The dose-response can be derived for an arbitrary number of reactions as long as the mass conservation equations are bilinear as in (3) [20,22]. Although, our reactions are reversible and obey detailed balance, the theory can also incorporate dissipative irreversible steps (see Methods) [36]. Since [*Y*_3_] is proportional to [*Y*_2_] and this is true for all concentrations following the CLS, a more general form for the activity is the sum of all of these concentrations. Biophysically, this implies that the final product can arise from each step after the CLS independently.

As shown in Fig. 1, decelerators can interact with accelerators. Consider the competitive decelerator *D* interacting with *X*_1_ via $X_{1}+D↔{X^{\prime }}_{1}$. If *D* is in excess, the addition of *D* leads to one additional equilibrium condition, $\left[{X^{\prime }}_{1}\right]=q^{\prime }[D][X_{1}]$, and a modification to the mass conservation law for *X*_1_ (3a) to $\left[X_{1}\right]+\left[Y_{1}\right]+\left[X^{\prime }\right]=X_{1}^{T}$. Solving the new equilibrium and mass conservation conditions then gives

$$\left[Y_{1}\right]=\frac{q_{1}X_{1}^{T}\left[Y_{0}\right]}{1+q_{1}\left[Y_{0}\right]+q^{\prime }\left[D\right]}$$

The equations for [*Y*_2_] and [*Y*_3_] are unchanged. Since [*Y*_1_] remains a linear-fractional function of [*Y*_0_] in the presence of inhibition by *D*, the dose-response will also remain a linear-fractional function.

Activity can also be repressed by a reaction following the third reaction, *Y*_*3*_+*X*_*4*_ *↔Y*_*4*_, which can suppress [*Y*_3_] by diverting the product to a less productive pathway. The new reaction changes mass conservation for *X*_2_ (3b) to $\left[X_{2}\right]+\left[Y_{2}\right]+\left[Y_{3}\right]+\left[Y_{4}\right]=X_{2}^{T}$ which gives

$$\left[Y_{2}\right]=\frac{q_{2}X_{2}^{T}\left[Y_{1}\right]}{1+q_{2}\left(1+q_{3}X_{3}^{T}\left(1+q_{4}X_{4}^{T}\right)\right)\left[Y_{1}\right]}$$

This is again linear-fractional, yielding

$$\left[Y_{2}\right]=\frac{q_{1}X_{1}^{T}q_{2}X_{2}^{T}\left[Y_{0}\right]}{1+q^{\prime }\left[D\right]+q_{1}\left[Y_{0}\right]+q_{2}\left(1+q_{3}X_{3}^{T}\left(1+q_{4}X_{4}^{T}\right)\right)q_{1}X_{1}^{T}\left[Y_{0}\right]}$$

which is inhibited by $X_{4}^{T}$. Since $\left[Y_{3}\right]=q_{3}X_{3}^{T}\left[Y_{2}\right]$ and $\left[Y_{4}\right]=q_{4}X_{4}^{T}\left[Y_{3}\right]$, the most general expression of the activity that preserves non-cooperativity is the sum: $A=\sum_{i=2}^{4}a_{i-1}[Y_{i}]$. The activity as a function of the agonist and any other factor is linear-fractional with the form

$$A=\frac{V[Y_{0}]}{1+W[Y_{0}]}$$ (4)

where

$$V=\frac{q_{1}X_{1}^{T}q_{2}X_{2}^{T}\left(a_{1}+a_{2}q_{3}X_{3}^{T}+a_{3}q_{3}X_{3}^{T}q_{4}X_{4}^{T}\right)}{1+q^{\prime }\left[D\right]},W=\frac{q_{1}+q_{2}\left(1+q_{3}X_{3}^{T}\left(1+q_{4}X_{4}^{T}\right)\right)q_{1}X_{1}^{T}}{1+q^{\prime }\left[D\right]}$$ (5)

V = A_max_/EC_50_ and W = 1/EC_50_ are linear-fractional functions of the equilibrium constants and the concentrations of all factors (total concentration of accelerators and free concentration of decelerators) in the system. From (4) and (5), we see that activity can be repressed by the action of either a decelerator or an accelerator, provided the latter acts after the CLS and the contribution to the activity from [*Y*_4_] is less than that from [*Y*_2_] and [*Y*_3_]. We can write formulas for V and W for an arbitrary number of cofactors. Their functional forms are distinguished by the types of the cofactors (i.e., accelerators or one of 6 types of decelerator) and where they act in relation to each other and the CLS. The formulas for *V* and *W* for all the possible combinations of 2 factors are calculated in Dougherty et al. [22] and shown in S1 Table. They are always linear fractional functions of each accelerator or decelerator. They can be used to make predictions of the mechanisms of added factors on the basis of the graphs of V and W vs. the cofactor just as the Lineweaver-Burk plot is used in enzyme kinetics.

Finally, it is commonly accepted that transcription factors sometimes form oligomers before they act [17]. Our theory can be generalized to include factors acting through oligomers, provided that they do so non-cooperatively. For example, suppose that an accelerator *X* first forms an oligomeric complex with another factor *Z* in the reaction $X+Z\overset{r}{↔}XZ$ prior to interacting with a product *Y*_*i-1*_ in a reaction $Y_{i-1}+XZ\overset{q}{↔}Y_{i}$ before the CLS. If *Z* is super-abundant compared to *X* then the concentration of the product as a function of the previous product has the non-cooperative form

$$\left[Y_{i}\right]=\frac{rqX^{T}\frac{\left[Z\right]}{1+r\left[Z\right]}\left[Y_{i-1}\right]}{1+rq\frac{\left[Z\right]}{1+r\left[Z\right]}\left[Y_{i-1}\right]}$$

Hence, for accelerators acting through a hetero-oligomer, the limited factor, *X*, acts as an accelerator in our current theory while the action of the superabundant factor, *Z*, saturates with sufficiently high [*Z*]. A decelerator could likewise act through a hetero-oligomer.

### Application of theory to gene repression

We apply the theory to steroid-regulated gene repression by observing that gene activity in induction can be repressed by other factors and still maintain a linear-fractional form. Hence, a linear-fractional dose-response (1) can arise in repression if the steroid-receptor complex (GR) acts as either a decelerator at any position or an accelerator after the CLS of a gene initiated by some other inducer. We compute the dose-response for steroid-regulated gene repression (1) by substituting the steroid-receptor complex (GR) (or some activated form of GR) into the formulas for V and W in the dose-response (4) for a gene activated by another inducer. Note that the experimentally measured dose-response of gene activity with respect to the inducer need not be linear-fractional for this theory to hold. What is required is that downstream steps where the cofactors and GR act have the linear-fractional property.

It is well known that steroid binding to GR follows Michaelis-Menten kinetics in terms of [S] [20]. Suppose that GR acts as D in the above example. We substitute

$$[D]=[GR]=\frac{G[S]}{K+[S]}$$

into Equation (5) to obtain

$$V=\frac{q_{1}X_{1}^{T}q_{2}X_{2}^{T}\left(a_{1}+a_{2}q_{3}X_{3}^{T}+a_{3}q_{3}X_{3}^{T}q_{4}X_{4}^{T}\right)(K+[S])[Y_{0}]}{K+[S]+q^{\prime }G\left[S\right]},W=\frac{\left(q_{1}+q_{2}\left(1+q_{3}X_{3}^{T}\left(1+q_{4}X_{4}^{T}\right)\right)q_{1}X_{1}^{T}\right)\left(K+[S]\right)[Y_{0}]}{K+[S]+q^{\prime }G\left[S\right]}$$

Substituting this into (4) and clearing the fractions results in an activity that is linear-fractional in [*S*], which we can write as

$$A=\frac{T([S])}{U([S])}=\frac{T(0)+T^{\prime }*[S]}{U(0)+U^{\prime }*[S]}$$ (4’)

where

$$\begin{array}{l} T=q_{1}X_{1}^{T}q_{2}X_{2}^{T}\left(a_{1}+a_{2}q_{3}X_{3}^{T}+a_{3}q_{3}X_{3}^{T}q_{4}X_{4}^{T}\right)(K+[S])[Y_{0}]\\ U=K+[S]+q^{\prime }G\left[S\right]+\left(q_{1}+q_{2}\left(1+q_{3}X_{3}^{T}\left(1+q_{4}X_{4}^{T}\right)\right)q_{1}X_{1}^{T}\right)\left(K+[S]\right)[Y_{0}] \end{array}$$

and the prime signifies derivative with respect to [*S*]. From this, we can thus surmise that

$$A_{max}=\frac{T(0)}{U(0)},A_{min}=\frac{T^{\prime }}{U^{\prime }},IC_{50}=\frac{U(0)}{U^{\prime }}$$

These three dose-response parameters are determined by the four quantities *T*(0), *U*(0), *T′*, and *U′* which implies that the combination parameter:

$$\frac{A_{max}IC_{50}}{A_{min}}=\frac{T(0)}{T^{\prime }}$$

is also linear-fractional. The theory predicts that these four dose-response parameters are always linear fractional and that there are four compatibility conditions between them: a) the numerator of A_max_ is equal to the numerator of A_max_×IC_50_/A_min_, b) the numerator of A_min_ is equal to the denominator of A_max_×IC_50_/A_min_, c) the denominator of A_max_ is equal to the numerator of IC_50_, and d) the denominator of A_min_ is equal to the denominator of IC_50_. These properties are not expected for arbitrary linear-fractional functions and provide a validity check for the theory.

Suppose we are only interested in the influence of an accelerator after the CLS (i.e., $X_{3}^{T}$ or $X_{4}^{T}$). We can then write T and U above in terms of the accelerator *X* ^*T*^, [*S*], and effective constants that depend on the parameters of the hidden reactions:

$$\begin{array}{l} T=(B_{1}+B_{2}X^{T})(K+[S])\\ U=K+[S]+q'G[S]+\left(B_{3}+B_{4}qX^{T}\right)\left(K+[S]\right) \end{array}$$

From which we immediately obtain

$$A_{max}=\frac{B_{1}+B_{2}X^{T}}{1+B_{3}+B_{4}X^{T}}$$ (6a)

$$A_{min}=\frac{B_{1}+B_{2}X^{T}}{1+q^{\prime }G+B_{3}+B_{4}X^{T}}$$ (6b)

IC50=(1+B+3B4XT)K1+q'G+B3+B4XT (6c)

$$\frac{A_{max}IC_{50}}{A_{min}}=K$$ (6d)

A cofactor can act like an accelerator or a decelerator before, at, or after the CLS. For two cofactors, such as GR and one other cofactor, there are 5 possible configurations for their action when not acting together at the same step (e.g., both before the CLS, one before and one at the CLS, etc.). There are 10 total positional combinations since GR can act before or after the other cofactor. There are 3 more configurations where GR acts at the same position as the other cofactor if one is a decelerator while the other is an accelerator. Thus, GR can act as a decelerator in all of these 13 configurations or as an accelerator after the CLS in 5 configurations. This gives a total of 18 possible configurations of GR and one other cofactor. A calculation for T and U can be made for each of these combinations and the results are in S2 Table. What these calculations show is how the dose-response parameters change as a function of differing amounts of added cofactor.

The experimental dose-response can be fit to the predictions for each of the 18 cases to see which fits best. However, many of the cases can be eliminated immediately based on qualitative properties of the curves. The dose-response parameters will always be linear-fractional functions with the form

$$y=\frac{a+bx}{c+dx}$$

Depending on the parameters, *y* can appear like a constant, a linear function, a Michaelis-Menten function, or a general linear-fractional function that increases or decreases with *x*. We also know that *y* increases with *x* if *ad<bc*, decreases if *bc<ad*, and is a constant if *ad = bc*. The *x* value for half-maximal *y* (half-maximal concentration) is c/d, and a/b for 1/*y*. Using these properties, we see that A_min_ and A_max_ in Equations (6a) and (6b) for the three reaction example can either increase or decrease depending on the parameter values but if A_max_ increases then so must A_min_. IC_50_ in (6c) is an increasing function because the denominator has an extra positive constant term. The graph of A_max_×IC_50_/A_min_ in (6d) is a horizontal line. Similar predictions for all the possible combinations of GR and one cofactor are summarized in Table 1. The graph properties in Table 1 represent some sufficient conditions for the predicted mechanisms and position of action and do not represent a comprehensive list of all possible predictions.

**Table 1:** Predicted mechanism based on dose-response parameter plots.

Entry	Plot properties of Parameter vs F	Predictions
1	A_max_ constant	1. F is any activity after or at GR and GR is A after CLS 2. F is U after CLS and GR is any activity after F
2	A_max_ linear increasing through the origin	1. F is A at CLS
3	A_max_ nonlinear increasing through the origin	1. F is A before CLS
4	A_max_ decreasing, approaches zero for infinite F(1/A_max_ is linear increasing)	1. F is C or L before or at the CLS and GR is A after CLS 2. F is C or L before or at the CLS and GR is D before or at CLS
5	A_max_ and A_min_ nonlinear increasing positive at true zero of F	1. F is A after CLS and GR is A after F or GR is D 2. F is PU or PN before or at CLS 3. F is C after CLS and GR is A after F or GR is D
6	A_max_ nonlinear decreasing does not approach zero for infinite F	1. F is A after CLS and GR is A after F or GR is D 2. F is PU or PN before or at CLS 3. F is C after CLS and GR is A after F or GR is D
7	A_max_ increasing and A_min_ decreasing	1. F is A after CLS andGR is A or C, after F
8	A_min_ linear increasing through the origin	1. F is A at the CLS
9	A_min_ nonlinear increasing through the origin	1. F is A before CLS
10	A_min_ positive at true zero of F	1. F is A after CLS 2. F is PU or PN, before or at CLS
11	A_min_ decreasing, approaches zero for infinite F(1/A_min_ linear increasing)	1. F is C, LU, or LN, before or at CLS
12	H of A_min_ < H of A_max_	1. F is A after CLS and GR is A after F 2. F is C or L, before CLSand GR is A after CLS
13	H of A_min_ > H of A_max_	1. F is A before CLS and GR is C or L, at F 2. F is A after CLS and GR is D before or at CLS 3. F is C after CLS and GR is A at F 4. F is C or L, at CLS, or F is C before CLS and GR is A after CLS
14	H of 1/A_min_ < H of 1/A_max_	1. F is A after CLS and GR is A after F
15	H of 1/A_min_ > H of 1/A_max_	1. F is C after CLS and GR is A at F
16	H of 1/A_min_ = H of 1/A_max_	1. F is A after CLS and GR is D before or at CLS
17	IC_50_ constant	1. F is A at the CLS
18	IC_50_ increases	1. F is L before or at CLS and GR is A after CLS 2. F is C and GR is A after CLS 3. F is A before CLS and GR is C or L, before CLS
19	IC_50_ decreases	1. F is A before or after CLS and GR is A after CLS 2. F is PU or PN before CLS and GR is A after CLS 3. F is C or L, before CLS and GR is C or L, before CLS
20	A_max_*IC_50_/A_min_ constant	1. Either F or GR acts before or at the CLS
21	A_max_*IC_50_/A_min_ increases	1. F is C after CLS andGR is A after CLS 2. F is A after CLS and GR is D after CLS 3. F is D after CLS and GR is D after F
22	A_max_*IC_50_/A_min_ decreases	1. F is A after CLS and GR is A after CLS 2. F is A after CLS and GR is D after F 3. F is D after GR and GR is D after CLS

The predictions are derived by examining the formulas for these parameters as shown in S2 Table. F means Factor, GR means steroid-receptor complex, A means accelerator, D means any type of decelerator, C means competitive decelerator, U means uncompetetive decelerator, N means noncompetitive decelerator, L means linear decelerator, and P means partial decelerator. H is the concentration for the half-maximum of the dose-response parameter. This table represents sufficient conditions and is not complete. Note that two cofactors cannot be of the same type at the same step, GR will not repress gene expression if it is an A before the CLS, and the A_min_ graphs allow GR to act anywhere as a D or as an A after the CLS.

### Experimental validation of the theory

Our experimental paradigm for steroid-regulated repression is the well-documented GR inhibition of phorbol myristate acetate (PMA) induction of a reporter construct (AP1LUC) with the human collagenase-3 promoter [15,28,29]. We usually measured the gene activity for four concentrations of the GR agonist Dex including EtOH in the presence of different concentrations of AP1LUC reporter plus one of three added cofactors: the plasmid for TIF2 or two small molecules (NU6027 and phenanthroline) recently identified in a high throughput screen as accelerators of GR transactivation [30].

**Fig 3:**
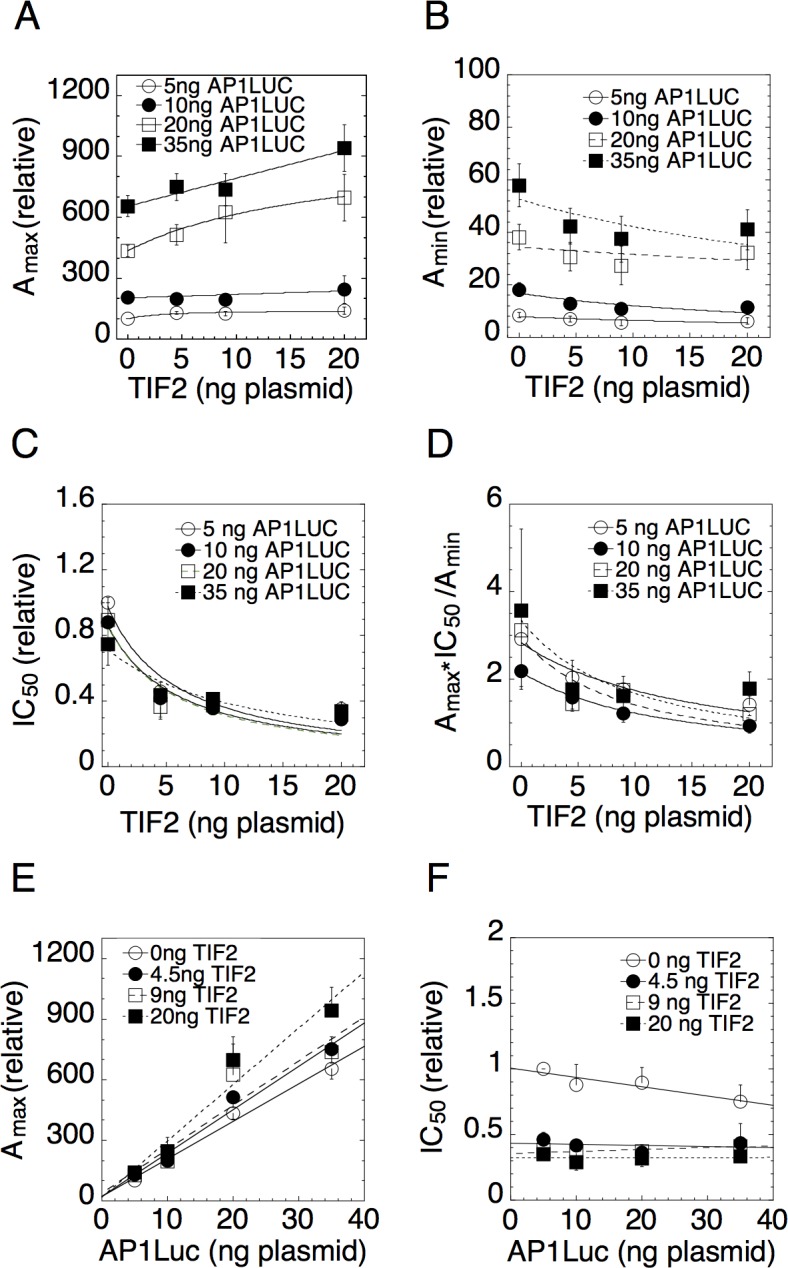
Plots of dose-response parameters for varying concentrations of AP1LUC and TIF2. Experiments were conducted with triplicate samples of U2OS.rGR cells that were transiently transfected with the indicated concentrations of AP1LUC and TIF2 plasmids and treated with 25ng/ml of PMA for four concentrations of Dex. Average plots vs. TIF2 for (A) A_max_, (B) A_min_, (C) IC_50_, and (D) A_max_×IC_50_/A_min_ and vs. AP1LUC for (E) A_max_ and (F) IC_50_ were obtained by first normalizing the data to the value for the lowest amount of AP1LUC and factor and then averaging and plotting the values (n = 4–12, ± S.E.M.).

**Fig 4:**
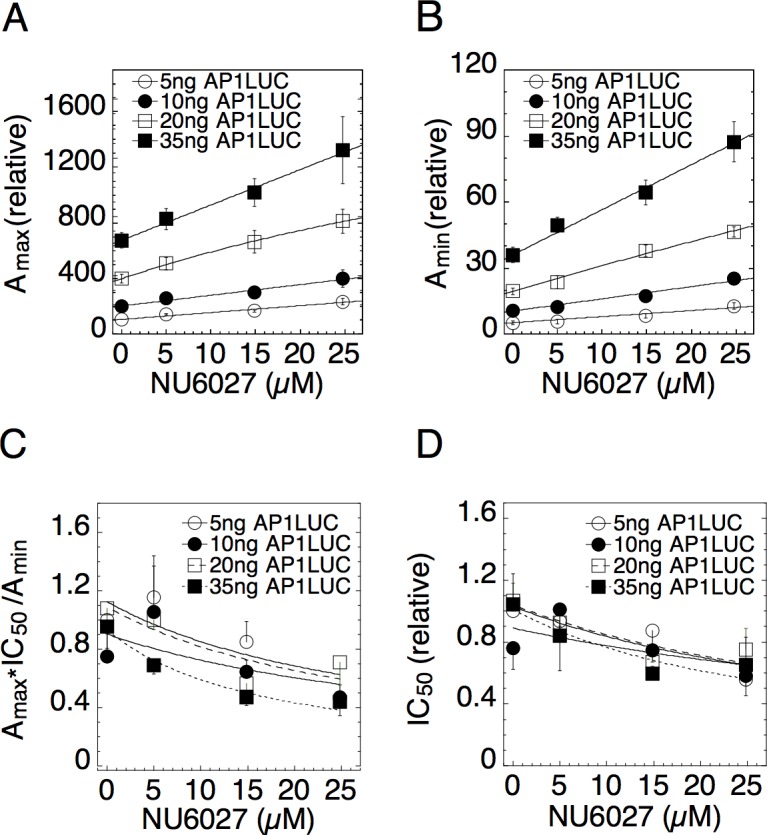
Dose-response parameters for varying concentrations of AP1LUC and NU6027. Experimental assays were conducted as in Fig. 3 with 10ng/ml of PMA and four concentrations of Dex. Average plots of (A) A_max_, (B) A_min_, (C) A_max_×IC_50_/A_min_, and (D) IC_50_ vs. NU6027 were obtained by first normalizing the data to the value for the lowest amount of AP1LUC and factor and then averaging and plotting the values (n = 5, ± S.E.M.).

**Fig 5:**
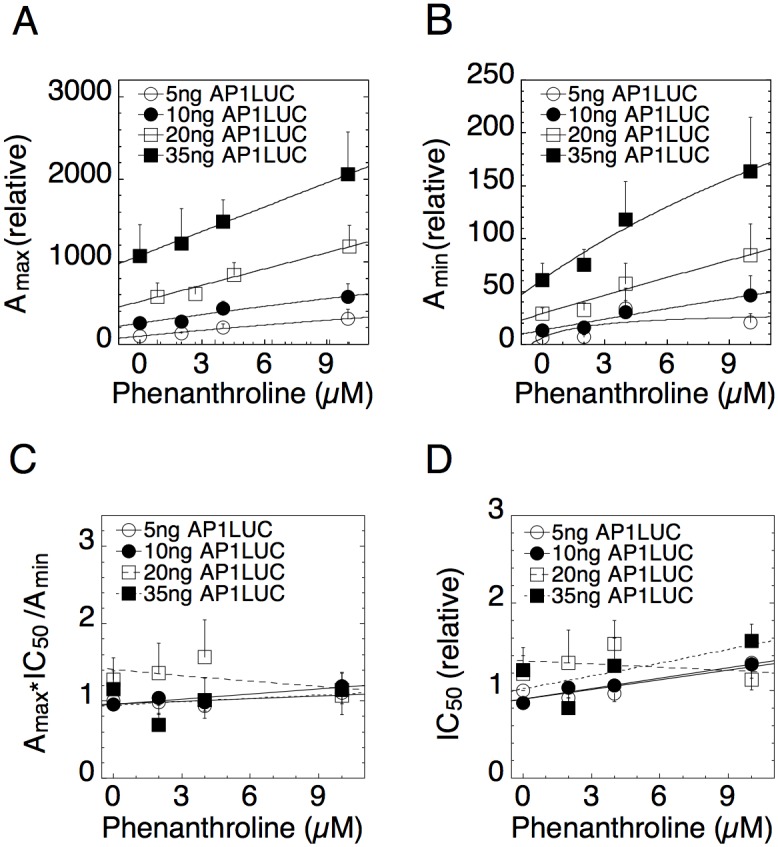
Dose-response parameters for varying concentrations of AP1LUC and phenanthroline. Experimental assays were conducted as in Fig. 4. Average plots of (A) A_max_, (B) A_min_, (C) A_max_×IC_50_/A_min_, and (D) IC_50_ vs. NU6027 were obtained by first normalizing the data to the value for the lowest amount of AP1LUC and factor and then averaging and plotting the values (n = 5, ± S.E.M.).

We present three lines of evidence to support the application and validity of the theory. 1) Fig. 2 shows the dose-response curve for GR repression of PMA induction of AP1LUC without (Fig. 2A) or with added TIF2 (Fig. 2B). The curves (including EtOH) are well fit by the linear-fractional function in Equation (1) (solid lines) as required by the theory. We found that excellent fits of the dose-response data to Equation (1) could be obtained with four points (3 concentrations of Dex plus EtOH) (R^2^ = 0.984 ± 0.026 [S.D., n = 160 randomly selected plots], median = 0.993), from which we estimated the dose-response parameters A_max_, A_min,_ and IC_50_ in the ensuing experiments. 2) Figs. 3–5 show plots of A_max_, A_min,_ IC_50,_ and A_max_×IC_50_/A_min_ determined from four steroid concentrations for varying amounts of AP1LUC reporter and each of the added cofactors. The parameters are all well fit by linear-fractional functions (solid curves) as predicted by the theory. 3) We tested if these parameter graphs satisfied the four predicted compatibility conditions using Bayesian model comparison as detailed in the Methods. We found that the Bayesian Information Criterion (BIC) is lowest for the predicted model compared to two null models (see Tables S3, S4, and S5), which further validates the theory. Given the confidence that the theory is applicable, we used it to make predictions for the mechanism and position of action for the added cofactors as well as for the reporter and GR.

### Mechanism and location of TIF2, NU6027, and phenanthroline

Since the dose-response for repression is derived from the dose-response for induction, how a factor affects the A_max_ produced by the inducer in GR-regulated repression should be the same as how it alters A_max_ in GR-regulated gene induction even if the inducer is different in the two cases. That this is so can be seen from the formulas for A_max_ in induction (S1 Table) and repression (S2 Table). In our system, the A_max_ in gene repression is the response to PMA alone and occurs *in the absence of added steroid*. All the cofactors we considered (other than the reporter) were found to be accelerators after the CLS in steroid-mediated gene induction [22,30]. The above example and S2 Table show that a graph of A_max_ vs. cofactor can inform us of where an accelerator acts in gene repression and these predictions are summarized in Table 1.

We used four concentrations of both AP1LUC and TIF2 in our competition assay to analyze TIF2 action in GR-regulated gene repression in U2OS.rGR cells. The graphs of A_max_, A_min,_ IC_50,_ and A_max_×IC_50_/A_min_ vs. TIF2 (Figs. 3A-D) all have linear-fractional shapes (solid lines) as predicted. The data for A_max_ vs. TIF2 (Fig. 3A) are well fit by Michaelis-Menten functions that have an x-axis intersection coordinate of -46.2 ± 26.8 ng (S.E.M., n = 4) of TIF2 plasmid. As is true in the competition assays for gene induction [22], the interpretation of the graphs for gene repression requires that the x-axis values reflect the total amount of factor, i.e., the sum of endogenous plus exogenous factor (also see Methods). From quantitative Western blots (not shown), it was determined (assuming 50% transfection efficiency of cells [22]) that the endogenous TIF2 is equivalent to 2.7 ± 1.5 ng (S.E.M., n = 3) of plasmid. Thus the point of zero endogenous TIF2 is at -2.7 ng TIF2 plasmid, which is much more positive than the intersection point of the curves at -46, despite the large error range. As seen in Equation (6a), S2 Table (since *T(0)>0* for $X^{T}=0$ for all entries where *k* > CLS), and summarized in Table 1 (entry 5), this is consistent with TIF2 acting as an accelerator after the CLS.

Figs. 4 and 5 show that the dose-response parameters are also well fit by linear-fractional functions for the small molecules NU6027 and phenanthroline [30]. A_max_ and A_min_ versus both cofactors are lines that intersect at values more negative than the concentration of endogenous chemicals, which is zero. This behavior is consistent with both compounds being accelerators after the CLS (Figs. 4A&B and 5A&B and entries 5 and 10 of Table 1). The conclusions for TIF2, NU6027 and phenanthroline being accelerators acting after the CLS in gene repression are consistent with what was observed in gene induction [30].

### Mechanism and location of the reporter AP1LUC

The traces in the graphs of A_max_ vs. AP1LUC (Fig. 3E) for varying concentrations of TIF2 are all linear, intersecting at the origin. The linear plot of Fig. 3E is preferred over a nonlinear plot (BIC = 46.39 vs. 55.56 respectively). As can be seen from the example above, in the formulas of S2 Table where k = CLS and summarized in Table 1 (entry 2), this is consistent with AP1LUC acting as an accelerator at the CLS. Graphs of IC_50_ vs. AP1LUC (Fig. 3F) consist of near horizontal lines (e.g., a constant slope) that decrease in position with added TIF2 (average slope = -0.0016 ± 0.0038, S.D., n = 4 traces of graph). These plots, summarized in Table 1 (entry 17), are also diagnostic of the reporter AP1LUC being an accelerator (A) at the CLS.

S1 A&B and S2 A&B Figs. show that A_max_ and A_min_ versus AP1LUC for varying concentrations of NU6027 and phenanthroline are again linear through the origin. The graphs of IC_50_ vs. AP1LUC have a constant zero slope with NU6027 (= -0.0009 ± 0.0064, S.D., n = 4 traces) and with phenanthroline (= 0.0067 ± 0.0070, S.D., n = 4 traces) (S1 C and S2 C Figs). These imply that AP1LUC is an accelerator at the CLS in the presence of both NU6027 and phenanthroline. Hence, we find that the reporter is always an accelerator at the CLS in both gene induction and repression.

### Mechanism and location of GR

Generally, in order to determine the action of a factor, one measures the response to changes of that factor. However, this was not possible with GR because we could only observe the robust repression needed for accurate graphical analyses with the high amounts of stably transfected GR in our experimental system. However, we can still deduce the mechanism and location of GR by comparing the responses to changes in the cofactors to the formulas in S2 Table to see which behaviors for GR are compatible with the observed results.

Fig. 3 indicates that A_max_ increases while A_min_, IC_50_ and A_max_×IC_50_/A_min_ all decrease vs. TIF2. As we show in the Methods, this is mathematically possible only if GR acts as an accelerator after TIF2 and TIF2 is an accelerator after the CLS. The results are also summarized in Table 1 (entries 7, 19 and 22). Furthermore, from our posterior parameter estimates of our Bayesian model comparison test (see S3 Table), we find that the concentration of TIF2 at half-maximal A_max_ (parameter 1) is greater than the same for A_min_ (parameter 3). This condition is also true for 1/A_max_ (parameter 2) and 1/A_min_ (parameter 4). These conditions further support the above deductions that TIF2 is an accelerator after the CLS and GR acts as an accelerator after both the CLS and TIF2 (Table 1, entries 12 and 14).

Figs. 4 and 5 show that A_max_ and A_min_ are each augmented by increasing concentrations of both NU6027 and phenanthroline. Hence, these cofactors cannot uniquely predict the action of GR. However, they can still be used to test for consistency. Figs. 4C&D show that A_max_×IC_50_/A_min_ and IC_50_ are both decreasing versus NU6027. According to entries 22 and 19 respectively of Table 1, these graphs support the inference that GR acts as an accelerator after NU6027, which acts as an accelerator after the CLS. The concentrations of NU6027 at half-maximal A_max_ and 1/A_max_ are larger than those of A_min_ and 1/A_min_ respectively (S4 Table), which is also consistent with the conclusion that NU6027 acts as an accelerator after the CLS and before GR.

Unlike TIF2 and NU6027, A_max_×IC_50_/A_min_ and IC_50_ versus phenanthroline do not exhibit any obvious trends (Figs. 5C&D). However, an examination of the formulas for A_max_×IC_50_/A_min_ and IC_50_ in S2 Table shows that there are parameter regimes where A_max_×IC_50_/A_min_ and IC_50_ vary so slowly that they would appear constant when the factor acts after the CLS. There was also no significant difference between the half-maximal concentrations of A_max_ and A_min_ and their reciprocals for phenanthroline (S5 Table, mean posteriors). Hence, these data neither confirm nor contradict the above conclusion that GR acts as an accelerator after phenanthroline.

**Fig 6:**
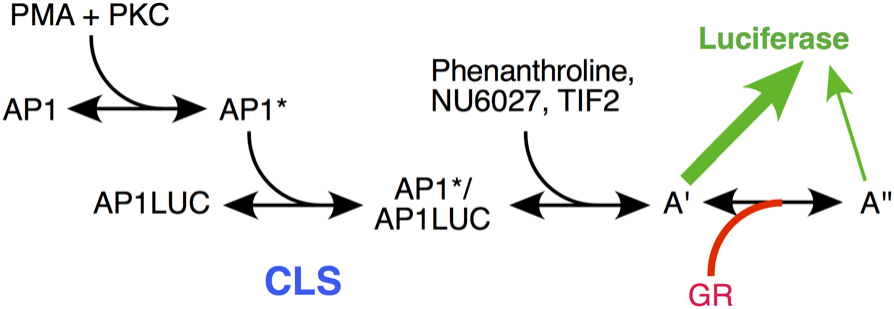
Predicted reaction scheme of PMA induction of Luciferase activity from synthetic reporter (AP1LUC) by AP1 that is repressed by steroid-bound receptor (GR). The position of the CLS, and positions of action of TIF2, NU6027, phenanthroline, and GR, as determined by the data of Figs. 3–5, are indicated. A’ and A” represent unknown, post-CLS steps, each of which can lead to Luciferase activity but the efficiency from A” is much less than A’.

Therefore, in this system, the reporter (AP1LUC) and the added cofactors display the same kinetically-defined mechanisms of action, and at the same positions relative to the CLS, in GR-regulated gene repression and gene induction (Fig. 6). The only difference is that the position, but not mechanism, of GR action changes in gene repression from that in gene induction.

## Discussion

We introduce a theory for GR-regulated gene repression that is based on first principles. The theory is general but is mathematically solvable only when the dose-response curves for gene repression are linear-fractional (Fig. 2). The theory accommodates any number of pathway steps, transcription factors, and cofactors that alter the A_max_, A_min_, and/or IC_50_ of GR-controlled gene repression. The formation of multicomponent complexes is permitted as long as their concentrations are low or their lifetimes are short, which is biologically reasonable and has been observed for numerous factors [33–35]. The theory could also be generalized to include the action of factors through preformed hetero-oligomeric complexes with other factors. The competition assay for gene repression, like that for GR transactivation [20,22,23,27], informs the kinetically-defined mechanism of action of each factor (i.e., accelerator vs. one of six decelerators) and the position of factor action relative both to the other competing factor and to the CLS, which again appears to be an invariant marker in the overall reaction sequence (see below). The theory makes specific predictions regarding the graphs of A_max_, A_min_, IC_50_, and A_max_×IC_50_/ A_min_ vs. one factor with increasing concentrations of a second factor (Table 1). The utility of the theory has been tested by examining the effects in the competition assay of four factors: AP1LUC reporter, p160 cofactor TIF2, and two recently identified pharmaceutical modulators of GR transactivation [30].

The theory assumes mass action kinetics in a well-mixed medium. It cannot account for stochastic effects of a single transcription event. However, the averaged amount of mRNA in imaging experiments of a single agonist-induced gene does follow Michaelis-Menten kinetics [31] and may be analyzable by stochastic models [32]. Understanding the connection between our theory and stochastic models is an important future step.

In every comparison, the AP1LUC reporter is found to act as an accelerator at the CLS. The CLS corresponds to that step where the concentration of the accelerator is limited compared to its binding affinity but the free concentration of accelerators in reactions after the CLS are in excess compared to their bound concentrations. The CLS can also be thought of as the step in the reaction sequence where an initially limited accelerator is replaced by another limited accelerator, such as GR being replaced by reporter gene. Thus, the species transmitting the input signal undergoes a “baton pass”, as in a relay race, to a new species at the CLS. In induction, the reporter gene also always, and uniquely, acts as an accelerator at the CLS [23,25–27]. Thus, reporter action at the CLS is an invariant signpost in both gene induction (with GREtkLUC) and gene repression (with AP1LUC) about which all other modulating factors and cofactors can be arranged. This leads us to predict that this will also be the case in future examples of gene repression.

Experiments with varying concentrations of GR cannot be performed in the present system because only the U2OS.rGR cells with high amounts of endogenous GR gave robust repression. Cells with very low levels of GR, which would permit adding increasing concentrations of GR, did not yield the large fold-repression needed for the high precision measurements of the competition assay (data not shown). Nevertheless, careful examination of the equations for the theory of repression revealed that the position and mechanism of GR action can be deduced from various graphical characteristics with respect to another cofactor. In this manner it can be determined that GR acts as an accelerator after both the CLS and each of the three factors (TIF2, NU6027, and phenanthroline). Therefore, the kinetically-defined mechanism of action of GR, like that of the other factors including the reporter, is again the same as that seen during gene induction. What has changed for GR is its position of action. In gene induction, GR acts before the CLS and every other factor so far examined [23,25–27]. In contrast, GR functions after the CLS and each of the factors studied here in gene repression. This is not unexpected. Something about GR action must change if the increased response by GR in gene induction is to become a decreased output in gene repression. Furthermore, as the induction of the AP1LUC reporter occurs with added PMA in the absence of GR, it is plausible that GR inhibition might occur at a step downstream of the induction reaction. In this respect, we note that GR represses TNFα induction of IL-8 gene expression by acting after transcription initiation [37]. An accelerator acting after the CLS can increase or decrease the total gene activity depending on the parameters. We propose that repression of AP1LUC results from GR acting as an accelerator to favor a less productive step after PMA-mediated induction, thereby decreasing the level of LUC expression (Fig. 6). It is important to realize that GR can be found to display identical mechanisms of action in induction and repression while interacting with different factors.

The mathematical models for both gene repression and gene induction by GR require the sequential binding of GR monomers as opposed to the binding of preformed dimers. This is supported by the observation of non-cooperative dose-response curves with unit Hill coefficient in both GR-mediated repression (Fig. 2) and induction [20]. The binding of preformed GR dimers would yield a dose-response curve with greater than unity Hill coefficient. The sequential binding of monomers in gene induction to form DNA-bound dimers is further supported by the activity of several dimerization-defective GR mutants [20] and by biophysical studies of other dimerizing proteins that are found to associate with DNA by the sequential binding of monomers to yield DNA-bound dimers [38–43]. Similar experiments with dimerization-defective GR mutants cannot be performed in the current system due to the weak response in cells with very low levels of endogenous GR. However, it currently appears that most, if not all, instances of GR-mediated repression may also involve dimers of GR that are bound or tethered to DNA [44,45]. We strongly suspect that, as in gene induction, the association of GR with the DNA of repressed genes also proceeds via the step-wise binding of GR monomers, as required by our mathematical model.

How to define the actions of cofactors for gene repression has been complicated by the fact that GR causes changes in total activity that are opposite those seen in gene induction [4–11,15]. Therefore, any classification of cofactor action relying on final activity is doomed to ambiguities. The present competition assay defines cofactor activity in an unbiased manner because it considers only what is happening at the position of cofactor action, independent of the direction of changes in final response. Such stepwise considerations can now be achieved independent of the biochemical processes involved and are essential if one desires to selectively modify GR-regulated repression (and induction) of specific genes.

Using a common set of definitions of cofactor action [23], we find that TIF2, NU6027, and phenanthroline are all accelerators after the CLS in both gene induction [21–25,30] and gene repression. Despite apparently identical mechanisms of TIF2, NU6027, and phenanthroline in GR-regulated gene repression, there are some graphical differences. This is due to a combination of where they act relative to each other and the specific reaction parameters, which cannot be defined exactly with our current data. Thus identical mechanisms of action do not require precisely identical graphs. Nonetheless, this permits great mechanistic simplifications. It suggests that at least those cofactors that are accelerators act in a constant and modular fashion, independent of the changes in the final product and the inducing agonist. Such common kinetically-defined mechanisms of cofactor action mean that new cofactor actions need not be invoked to account for the different responses in gene induction and repression because the underlying mechanisms are, in fact, the same. It also suggests that manipulation of cofactor action in induction vs. repression can simultaneously affect both pathways.

There have been many descriptions of binding partners for TIF2 under conditions where TIF2 reverses a transcriptional response [46–50]. However, it should be realized that those species to which a factor initially binds, or chemically modifies, are unlikely to constitute the position at which factor action is exerted, which is what is revealed by our competition assay. For example, paused RNA polymerase II action occurs at steps downstream from its initial binding [51,52] while protein modifications, such as histone acetylation, elicit their effects after the modifying protein has bound. Similarly, the involvement of different cofactor domains in, for example, repression vs. induction, does not require different mechanisms of action. Different domains may alter the strength of a specific cofactor-target interaction or cause similar modifications of different downstream targets [37,53,54]. Alternatively, the relative importance of two positions of cofactor action may be influenced by domain composition [25,26].

In summary, we have described a theoretical model for GR-mediated gene repression that defines factors by their action at a particular step of the overall reaction scheme, as opposed to the final outcome. The theory has been validated by experimental results with five factors. The mechanism and position of action of four factors is qualitatively identical to that previously found in gene induction. The difference is that the fifth factor, GR, is predicted to act before the CLS and various cofactors in gene induction and after the CLS and cofactors in gene repression, presumably by diverting the reaction scheme to, and accelerating, a less productive pathway. The apparent constancy of factor mechanism of action in gene induction and repression means that altering cofactor activities is predicted to simultaneously affect both GR-regulated pathways. Thus, new and uncharacterized pathways will not have to be considered, which will simplify approaches for maximizing desired outcomes. Finally, as for the theory for gene induction [20,22], the current theory for gene repression is general for any gene induction and gene repression process displaying a linear-fractional dose-response and thus could be of use to analyze the mechanisms of other inducible transcription factors.

## Methods

### Experiments

Unless otherwise indicated, cell growth was at 37°C and all other operations were performed at 0°C.

### Chemicals

Dexamethasone (Dex), PMA (phorbol myristate acetate), NU6027, and phenanthroline were purchased from Sigma (St. Louis, MO). Restriction enzymes and T4 DNA ligase were from New England Biolabs (Beverly, MA) and the dual-luciferase reporter assay was from Promega (Madison, WI).

### Plasmids

TIF2 (Hinrich Gronemeyer, IGBMC, Strasbourg, France), and AP1Luc (Inez Rogatsky, Weill Medical College, Cornell University) were generously donated.

### GR repression of AP1LUC in U2OS.rGR cells

U2OS.rGR cells were grown in DMEM media supplemented with 10% FBS and 0.1 mg/ml G418 and seeded at 30,000 cells per well in a volume of 300 μl per well in 24-well plates as previously described [29] with the following modifications. One day after seeding in 24-well plates, cells in FBS-free DMEM were transfected with reporter (AP1Luc), 10 ng of phRG-TK Renilla (Promega) as an internal control, and the indicated amounts of plasmids for various factors in OPTIMEM plus XTREME Gene HP (Roche; 0.8 μl/well). Four hours after transfection, cells were refed with DMEM/10% FBS. The next day, cells were treated with PMA (10–25 ng/ml) and various dilutions of dexamethasone (Dex) and chemical. Sixteen hours later, the cells were lysed in lysis buffer and assayed for reporter gene activity using dual luciferase assay reagents according to the manufacturer’s instructions (Promega, Madison, WI). Luciferase activity was measured by a GloMax® 96 Microplate Luminometer (Promega, Madison, WI). The data were normalized to Renilla TK luciferase activity and expressed as a percentage of the maximal response with Dex before being plotted ± S.D. unless otherwise noted.

### Two-factor competition assays

The basic protocol for gene induction [22,23] was followed except as noted in the text for 4x4 (all 16 combinations of 4 concentrations of both factor 1 and factor 2) assays with four concentrations of Dex, all in triplicate, for a total of 196 wells. All plots of the data assume a linear increase in factor plotted on the x-axis. When Western blots reveal a nonlinear relationship between the optical density of scanned protein band and the amount of transfected plasmid at constant levels either of total cellular protein, or of β-actin, the linear equivalent of expressed plasmid must be determined as previously described [22] (see also below).

### Correction for nonlinear protein expression

The nonlinear plot of OD vs. ng of transfected plasmid is first fit to the Michaelis-Menten formula

$$A_{max}=m1*plasmid/\left(m2+plasmid\right)$$

to obtain constants m1 and m2. The functional equivalent of the transfected plasmid that gives a linear OD vs. plasmid plot is then obtained from the formula of

Plasmid(linear)=m2*plasmid/(m2+plasmid)

The x-axis value of amount of plasmid in the various graphs is then this “corrected plasmid” value.

### General conditions for Concentration Limited Steps (CLS)

A linear-fractional dose-response arises when the mass conservation equations are bilinear. The general bilinear form for an arbitrary number of reactions is:

$$\begin{array}{c} {}[X_{1}]+[Y_{1}]=X_{1}^{T}\\ {}[X_{2}]+[Y_{2}]=X_{2}^{T}\\ \vdots \end{array}\}\text{ Pre-CLS}$$

$$[X_{j}]+[Y_{j}]+[Y_{j+1}]+\cdots +[Y_{k}]=X_{j}^{T}\text{ CLS}$$

$$\begin{array}{c} {}[X_{j+1}]=X_{j+1}^{T}\\ \vdots \\ {}[X_{k}]=X_{k}^{T} \end{array}\}\text{ Post-CLS}$$

$$\begin{array}{c} {}[X_{k+1}]+[Y_{k+1}]=X_{k+1}^{T}\\ \vdots \end{array}\}\text{ Pre-CLS}$$

$$[X_{l}]+[Y_{l}]+[Y_{l+1}]+\cdots =X_{l}^{T}\text{ CLS}$$

$$\begin{array}{c} {}[X_{l+1}]=X_{l+1}^{T}\\ \vdots \end{array}\}\text{ Post-CLS}$$

where there can be multiple CLS steps. The bilinearity of these equations is easily confirmed by recursively substituting the equilibrium conditions for the products. The reactions can be divided into pre-CLS, CLS, and post-CLS steps. In pre-CLS steps, only the accelerator and its immediate product appear in the mass conservation equation. This can be achieved if all downstream products have much lower concentration than the accelerator and its product, i.e., [*Y*_*j*_]<<[*Y*_*i*_], [*X*_*i*_], *j>i*, which is satisfied if [*Y*_*i+1*_]<<[*Y*_*i*_], [*X*_*i*_], i.e., products become successively smaller. Substituting in the equilibrium conditions gives $q_{i+1}X_{i+1}^{T}[Y_{i}]<<[Y_{i}]$ or $q_{i+1}X_{i+1}^{T}<<1$. Hence, accelerators are limited with respect to their affinities at pre-CLS steps and the CLS is the last step of this sequence for which the accelerator is limited. At post-CLS steps, the free concentration of accelerator is equal to the total concentration, i.e., the bound concentration is negligible. This is satisfied if [*Y*_*j*_]<<[*X*_*i*_], *j≥i*, which implies that the concentration of accelerator is in excess compared to the products and the reactions are pseudo-first order.

### Equivalent irreversible kinetic scheme for the theory

The crucial point for preserving linear-fractional dose-response is that the equilibrium conditions have the form of (2) and the mass conservation conditions have bilinear form. Any kinetic scheme, reversible or irreversible, that obeys a similar set of equations at steady state will have a linear-fractional dose-response. For example, consider the irreversible reaction *P*+*X* →*P′* + *X* and *P′*→ *P*. In this “hit-and-run” scheme, the accelerator *X* interacts transiently with *P* and modifies it. The modified *P* then relaxes back to the original state as a first order kinetic process. This reaction could occur for example if *P* is in an “excited” state, *X* nudges it to a lower energy state *P′*, and energy is expended to pump *P′* back to the excited state. The concentrations have kinetics

$$\frac{d[P^{\prime }]}{dt}=k_{f}[P][X]-k_{r}[P^{\prime }],\frac{d[P]}{dt}=-k_{f}[P][X]+k_{r}[P'],\frac{d[X]}{dt}=0$$

which has a steady state solution [*P*′] = *q*[*X*][*P*], where *q = k*_*f*_ */k*_*r*_, and conservation equations [*P*]+[*P′*] = *P* ^*T*^, [*X*] = *X* ^*T*^. The equations combine to yield *[P′] =* *q* *X* ^*T*^[*P*], thereby mimicking a post-CLS reaction.

Conversely, we could have the irreversible reaction *P*+*X* →*X′* + *P* and *X′*→ *X*, where the product from the previous reaction now transiently interacts with an accelerator and modifies it to create a new product *X′*. In steady state, the new product obeys [*X′*] = *q*[*X*][*P*] with mass conservation condition [*X*]+[*X′*] = *X* ^*T*^, or [*X*]+*q*[*X*][*P*] = *X* ^*T*^. The equations combine to yield

$$[X^{\prime }]=\frac{qX^{T}[P]}{1+q[P]}$$

If this reaction were followed by a reaction of the form *X*′+*U*→*X*″+*U*, *X*″→ *X*′ then the mass conservation law for *X* changes to [*X*]+[*X′*]+[*X*″] = *X* ^*T*^. Combining with the additional steady state equation [*X″*] = *q′U* ^*T*^[*X*′], we obtain

$$[X^{\prime }]=\frac{qX^{T}[P]}{1+q[P]+qq^{\prime }U^{T}[P]}$$

which mimics a CLS step. Hence, in this irreversible hit-and-run scheme, accelerators that successively modify a product are akin to post-CLS steps and a reaction where the product switches roles and modifies a factor to make a new product is akin to a CLS. Reversible and irreversible reactions could be combined as long as the steady state conditions resemble the equilibrium conditions (2) and the mass conservation conditions have bilinear form.

### Determining where GR acts by comparing models to TIF2 data

Experiments find that A_max_ increases while A_min_ decreases with an increase in TIF2, which acts as an accelerator after the CLS. Experiments also find that IC50 and A_max_×IC_50_/ A_min_ decrease with increases in TIF2. We show below that this behavior puts severe constraints on the possible mechanisms and positions where GR can act. Specifically, we need to determine for which cases it is possible for A_max_ > A_min_, and A_max_ increases while A_min_, IC_50_ and A_max_×IC_50_/ A_min_ all decrease for increases of a post-CLS accelerator. We examine each possible case for TIF2 acting after the CLS individually.

#### 1. GR is a decelerator before the CLS

The dose-response parameters are given in S2 Table, entry 5) where the concentration of TIF2 is represented by *X* ^T^. Since GR acts before the CLS, A_max_×IC_50_/ A_min_ is a constant, which is ruled out by the experimental data. Hence, GR cannot be a decelerator before the CLS.

#### 2. GR is a decelerator at the CLS

From S2 Table, entry 8 we find that A_max_×IC_50_/ A_min_ is a constant, which rules this case out. Additionally, we find that

$$\begin{array}{l} A_{max}=\frac{B_{1}+B_{2}X^{T}}{(1+B_{3})+B_{4}\left(B_{5}+B_{6}X^{T}\right)q}\\ A_{min}=\frac{\left(B_{1}+B_{2}[X]\right)(1+\alpha \beta q^{\prime }G)}{(1+B_{3})(1+\gamma q^{\prime }G)+B_{4}\left((B_{5}+B_{6}X^{T})+\alpha q^{\prime }G\right)q} \end{array}$$

where *X* ^T^ is a post-CLS accelerator representing TIF2 and we have redefined parameters to suppress irrelevant ones. For A_max_ to increase and A_min_ to decrease with TIF2, we must satisfy the inequalities

$$\begin{array}{l} B_{2}\left(1+B_{3}+B_{4}B_{5}\right)>B_{1}B_{4}B_{6}\\ B_{2}\left(1+B_{3}+B_{4}B_{5}\right)+B_{2}\left(\left(1+B_{3}\right)\gamma +\alpha \right)q^{\prime }G<B_{1}B_{4}B_{6} \end{array}$$

These can never be satisfied simultaneously since all parameters are positive, thus further ruling out GR at the CLS.

#### 3. GR is an accelerator after the CLS but before TIF2

From S2 Table entry 18, we find that

$$A_{max}=\frac{B_{0}}{1+B_{3}}$$

Hence, A_max_ does not depend on the accelerator, which makes this case impossible since A_max_ increases with increased TIF2.

#### 4. GR is a competitive decelerator after the CLS but before TIF2

From S2 Table entry 13, we have after redefining parameters

$$A_{max}=\frac{B_{0}+B_{1}+B_{2}X^{T}}{1+B_{3}+B_{4}+B_{5}X^{T}},A_{min}=\frac{B_{0}\left(1+\gamma q^{\prime }G\right)+B_{1}+B_{2}X^{T}}{\left(1+B_{3}\right)\left(1+\gamma q^{\prime }G\right)+B_{4}+B_{5}X^{T}}$$

For A_max_ to increase while A_min_ decreases we must satisfy

$$0<B_{2}\left(1+B_{3}\right)-B_{0}B_{5}+B_{2}B_{4}-B_{5}B_{1}<\left(B_{0}B_{5}-B_{2}(1+B_{3})\right)\gamma q^{\prime }G$$ (M1)

For A_max_ > A_min_ we require

$$B_{1}(1+B_{3})-B_{0}B_{4}>(B_{0}B_{5}-B_{2}(1+B_{3}))X^{T}$$

To satisfy the second inequality in (M1) we require *B*_*0*_ *B*_*5*_ *>B*_*2*_(1+*B*_*3*_) and G sufficiently large. Without loss of generality we can let *Z* = *B*_1_(1+*B*_3_)/*B*_0_ and *Y* = *B*_2_(1+*B*_3_)/*B*_0_ since *B*_0_ > 0. Hence

$$0<Y-B_{5}+B_{4}Y/(1+B_{3})-B_{5}Z/(1+B_{3})<\left(B_{5}-Y\right)\gamma q^{\prime }G$$ (M2)

$$Z-B_{4}>(B_{5}-Y)X^{T}$$ (M3)

In the best-case scenario, we can set *X* ^T^ = 0 and let *B*_*4*_ = *Z* − *η*, where *η* >0 to satisfy (M3). Likewise we can set *B*_*5*_ = *Y*+*k*, where *k* > 0 to satisfy the second inequality of (M2). Substituting into the first inequality of (M2) then yields 0< −*k* − η *Y* / (1+*B*_*3*_) − *kZ*/(1+*B*_*3*_) which cannot be satisfied for positive parameters. Hence, GR cannot be before TIF2.

#### 5. GR is an accelerator after TIF2

From S2 Table, entry 16, we have after rescaling

$$A_{max}=\frac{B_{0}+B_{1}X^{T}}{1+B_{3}+B_{4}X^{T}},A_{min}=\frac{B_{0}+\left(B_{1}+B_{2}qG\right)X^{T}}{1+B_{3}+\left(B_{4}+B_{5}qG\right)X^{T}}$$

For A_max_ > A_min_, we must satisfy

$$(B_{0}+B_{1}X^{T})B_{5}>(1+B_{3}+B_{4}X^{T})B_{2}$$ (M4)

For A_max_ to increase and A_min_ to decrease we require *B*_*1*_(1+*B*_*3*_) − *B*_*4*_ *B*_*0*_ *>* 0, (*B*_*1*_ *+ B*_*2*_ *qG*)(1+*B*_3_)-(*B*_4_+*B*_*5*_ *qG)B*_*0*_<0, which can be rewritten as

$$0<B_{1}(1+B_{3})-B_{4}B_{0}<(B_{0}B_{5}-B_{2}(1+B_{3}))qG$$ (M5)

But from (M4) we obtain *B*_*0*_ *B*_*5*_ *- B*_*2*_(1+*B*_*3*_)> (*B*_*2*_ *B*_*4*_ *- B*_*1*_ *B*_*5*_ *)X* ^*T*^, which is guaranteed if *B*_*0*_ *B*_*5*_ *> B*_*2*_(1+*B*_*3*_) and *B*_*2*_ *B*_*4*_ *< B*_*1*_ *B*_*5*_. Thus the second inequality in (M5) can be satisfied for *G* sufficiently large. The last condition we require is that *B*_*4*_ *B*_*0*_ *< B*_*1*_(1+*B*_*3*_). These three conditions can be satisfied for *B*_1_ and *B*_5_ sufficiently large and *B*_0_ is not zero. A_min_ can be made to decrease if *B*_3_ is made sufficiently large. Hence, there are enough degrees of freedom to satisfy all the constraints and allow A_min_ to increase or decrease with a change of a single parameter. Hence, GR acting as an A after the CLS and TIF2 is a possible candidate mechanism.

We can examine this further by considering A_max_ and A_min_ with the other two dose-response parameters:

$$IC_{50}=\frac{K\left(1+B_{3}+B_{4}X^{T}\right)}{1+B_{3}+\left(B_{4}+B_{5}qG\right)X^{T}},\frac{A_{max}IC_{50}}{A_{min}}=\frac{K\left(B_{0}+B_{1}X^{T}\right)}{B_{0}+\left(B_{1}+B_{2}qG\right)X^{T}}$$

We find that A_max_ and A_min_ are linear-fractional functions that never go through the origin. A_max_×IC_50_/A_min_ and IC_50_ are necessarily decreasing functions of *X* ^T^. Additionally, the value of *X* ^T^ at half-maximal A_max_ is always greater than that of A_min_. This is also true of 1/A_max_ and 1/A_min_. These behaviors are consistent with the data for TIF2.

#### 6. GR is a competitive decelerator after TIF2

From S2 Table entry 9 we have after redefining parameters:

$$A_{max}=\frac{B_{0}+\left(B_{1}+B_{2}\right)X^{T}}{1+B_{3}+\left(B_{4}+B_{5}\right)X^{T}},A_{min}=\frac{B_{0}\left(1+\gamma q^{\prime }G\right)+\left(B_{1}(1+\gamma q^{\prime }G)+B_{2}\right)X^{T}}{\left(1+B_{3}\right)\left(1+\gamma q^{\prime }G\right)+\left(B_{4}(1+\gamma q^{\prime }G)+B_{5}\right)X^{T}}$$

For A_max_ > A_min_, we must satisfy *B*_*2*_(1+*B*_*3*_) − *B*_*0*_ *B*_*5*_ > (*B*_*1*_ *B*_*5*_ *- B*_*2*_ *B*_*4*_ *)X* or *Y> (B*_*1*_ *B*_*5*_ *- B*_*2*_ *B*_*4*_ *)X* where *Y = B*_*2*_(1+*B*_*3*_) − *B*_*0*_ *B*_*5*_. For A_max_ to increase, we must satisfy *B*_*2*_(1+*B*_*3*_) − *B*_*0*_ *B*_*5*_ *- (B*_*0*_ *B*_*4*_ *-B*_*1*_(1+*B*_*3*_)) > 0 or *Y* − *Z* > 0 where *Z* = *B*_*0*_ *B*_*4*_ *- B*_*1*_(1+*B*_*3*_). Likewise for A_min_ to decrease, we must satisfy *W* − *Z < Zγq′G*. Hence, these conditions are satisfied if we choose *B*_*1*_ *B*_*5*_ *- B*_*2*_ *B*_*4*_ *<* 0, *W* > *Z* > 0 and *γq′G* is large enough. There are enough degrees of freedom to satisfy these conditions. A_min_ can be made to increase by making Z small enough. Hence, GR could be a decelerator after the CLS and after TIF2 for A_max_ to increase and A_min_ to decrease.

The other two dose-response parameters are:

$$\begin{array}{l} IC_{50}=\frac{1+B_{3}+\left(B_{4}+B_{5}\right)X^{T}}{\left(1+B_{3}\right)\left(1+\gamma q^{\prime }G\right)+\left(B_{4}(1+\gamma q^{\prime }G)+B_{5}\right)X^{T}}\\ \frac{A_{max}IC_{50}}{A_{min}}=\frac{B_{0}+\left(B_{1}+B_{2}\right)X^{T}}{\left(1+B_{3}\right)\left(1+\gamma q^{\prime }G\right)+\left(B_{4}(1+\gamma q^{\prime }G)+B_{5}\right)X^{T}} \end{array}$$

We see immediately that IC_50_ increases for *γ* > 0. The value of *X* ^*T*^ at half-maximal of A_min_ is greater than that of A_max_ and similarly for 1/A_min_ and 1/A_max_. These behaviors are not consistent for TIF2. Hence, only one case is consistent with the data and we thus conclude that GR acts as an A after the CLS and after TIF2.

### Statistical analysis

Unless otherwise noted, all experiments were performed in triplicate multiple times. KaleidaGraph 4.1 (Synergy Software, Reading, PA) was used to determine a least-squares best fit (R^2^ was almost always 0.95) of the experimental data to the theoretical dose-response curve, which is given by the Equation (1). This was done by first estimating A_max_ directly from the data for zero Dex. The data points were subtracted from A_max_ and the resulting curve was then fit to a Michaelis-Menten function from which A_min_ and IC_50_ were determined. The values of *n* independent experiments were normalized, averaged, and then plotted and analyzed as described in the Supporting Information. The Bayesian Information Criterion was used to determine whether the best fit of A_max_ vs. AP1LUC data is obtained with linear or Michaelis-Menten plots.

The theory predicts that the four dose-response parameters are linear-fractional functions of the cofactor and satisfy four compatibility conditions. To test this, we fitted the theoretically predicted model, with two other “null” models to the data and used the Bayesian Information Criterion to see which model was best. The models tested were

1) Predicted Model:
  $$A_{max}=a_{5}[R]\frac{a_{1}+[X]}{a_{2}+[X]},A_{min}=a_{6}[R]\frac{a_{3}+[X]}{a_{4}+[X]},IC_{50}=a_{7}\frac{a_{2}+[X]}{a_{4}+[X]},A_{max}=a_{8}\frac{a_{1}+[X]}{a_{3}+[X]}$$
2) Permuted Model:
  $$A_{max}=b_{5}[R]\frac{b_{1}+[X]}{b_{4}+[X]},A_{min}=b_{6}[R]\frac{b_{2}+[X]}{b_{3}+[X]},IC_{50}=b_{7}\frac{b_{3}+[X]}{b_{2}+[X]},A_{max}=b_{8}\frac{b_{4}+[X]}{b_{1}+[X]}$$
3) Unconstrained linear-fractional model:
  $$A_{max}=c_{9}[R]\frac{c_{1}+[X]}{c_{5}+[X]},A_{min}=c_{10}[R]\frac{c_{2}+[X]}{c_{6}+[X]},IC_{50}=c_{11}\frac{c_{3}+[X]}{c_{7}+[X]},A_{max}=c_{12}\frac{c_{4}+[X]}{c_{8}+[X]}$$

where [*X*] is the concentration of the added cofactor, the *a*’s, *b*’s, *c’s* are parameters to be fitted to the data, and [*R*] is the concentration of the reporter, which we know is an accelerator at the CLS. The predicted and permuted models have the same model complexity with 8 free parameters, while the unconstrained model has 12 free parameters. The predicted model is a subset of the unrestricted model. The half maximum of the dose-response parameters are given by the parameter in the denominator. For example, the cofactor concentration for half maximum for the predicted model of A_max_ is given by *a*_2_, and that of A_min_ is given by *a*_4_. Conversely, the concentrations of half maximum of 1/A_max_ and 1/A_min_ are given by *a*_1_ and *a*_3_, respectively.

We used a Metropolis-Hastings Markov Chain Monte Carlo (MCMC) method [55] to compute the Bayesian posterior probabilities of the various parameters. The likelihood function we used was proportional to exp(−*χ* ^2^/2) where

$$\chi^{2}=-\sum_{p=1}^{k}\sum_{i=1}^{n}\frac{{\left(y_{i,p}^{\text{data}}-y_{i,p}^{\text{model}}\right)}^{2}}{\sigma_{i}^{2}}$$

The sum is over all n data points *i* for each dose-response parameter *p* out of *k* total, and the error variance $\sigma_{i,p}^{2}$ is the experimentally determined standard error variance of replicate experiments. The results for the maximum-likelihood values, and the mean and standard deviation for posteriors for TIF2, NU6027, and phenanthroline are in Tables S3 to S5. The reported results are for 2×10^7^iterations for the models with 8 parameters and 3×10^7^ for the unrestricted model after an even longer transient period to ensure convergence. The longer time for the unrestricted model was to compensate for the extra parameters. We used the Bayesian Information Criterion, BIC = *x* ^2^ + *k* ln *n*, to test which model best fit the data accounting for model complexity.

## Supporting Information

S1 Fig Dose-response parameters vs. varying concentrations of AP1LUC with added NU6027. Experimental assays were conducted as in Fig. 4 with 10ng/ml of PMA and four concentrations of Dex. Average plots of (A) A_max_, (B) A_min_, (C) IC_50_ vs. AP1LUC were obtained by first normalizing the data to the value for the lowest amount of AP1LUC and factor and then averaging and plotting the values (n = 5, ± S.E.M.).
(PDF)

S2 Fig Dose-response parameters vs. varying concentrations of AP1LUC with added phenanthroline. Experimental assays were conducted as in Fig. 5 with 10ng/ml of PMA and four concentrations of Dex. Average plots of (A) A_max_, (B) A_min_, and (C) IC_50_ vs. AP1LUC were obtained by first normalizing the data to the value for the lowest amount of AP1LUC and factor and then averaging and plotting the values (n = 5, ± S.E.M.).
(PDF)

S1 TableGene induction components in Equation (2) where $X_{i}^{T}$ means total accelerator concentration at some step *i*:, [*D*_*i*_] means decelerator concentration at some step *i*:, *α* = 0 for *competitive* inhibition, *γ = 0* for *uncompetitive* inhibition, *α* = *γ* for *noncompetitive* inhibition, *q* and *q*′are equilibrium or affinity constants.ε is a constant that is either 0 or 1. *B* parameters are positive constants that can take different values depending on context. These equations are derived in Dougherty et al. [22].(DOCX)

S2 Table Dose-response parameter components for various cases of GR and a factor. GR acts at location *g* and the factor acts at location *k*. GR as a repressor can act as either a decelerator anywhere or an accelerator after the CLS. The activated GR depends on steroid as [GR*] = *G*[*S*]/(*K*+[*S*]). Other parameters have the same meaning as in S1 Table. *B* parameters can have different values depending on context. The dose-response parameters are given by *A*_*max*_ *= T*(0)/*U*(0), *A*_*min*_ *= T*′/*V*′, *IC*_*50 =*_ *T*(0)/*T*′, and *A*_*max*_ *IC*_*50*_/*A*_*min*_ = *T(0)T*′
(DOCX)

S3 Table MCMC model fit results for TIF2. (DOCX)

S4 Table MCMC model fit results for NU6027. (DOCX)

S5 Table MCMC model fit results for Phenanthroline. (DOCX)

S1 Data Excel files of raw data. (ZIP)

S1 Text Steps involved in running competition assays for gene repression. (DOC)
