# Supplementary figures and images for: Kinetically-Defined Component Actions in Gene Repression
Source: PLoS Comput Biol. 2015 Mar 27;11(3):e1004122. doi: 10.1371/journal.pcbi.1004122 (PMC4376387; doi:10.1371/journal.pcbi.1004122)

Fig. S1

A

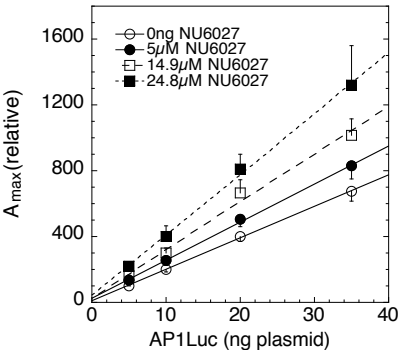

B

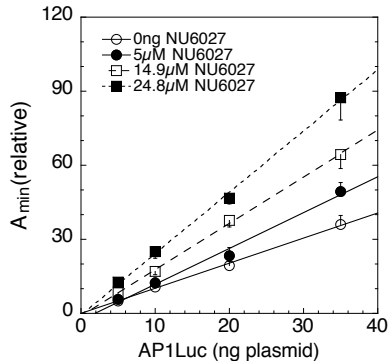

C

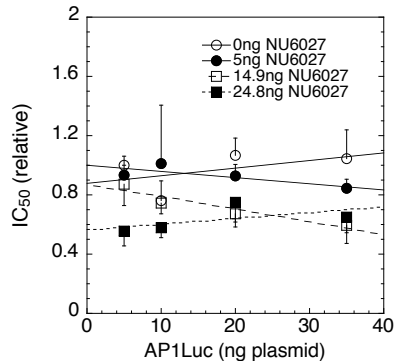

Supplement: S1 Fig — Experimental assays were conducted as in Fig. 4 with 10ng/ml of PMA and four concentrations of Dex. Average plots of (A) Amax, (B) Amin, (C) IC50 vs. AP1LUC were obtained by first normalizing the data to the value for the lowest amount of AP1LUC and factor and then averaging and plotting the values (n = 5, ± S.E.M.). (PDF) [file pcbi.1004122.s001.pdf]

Fig. S2

A

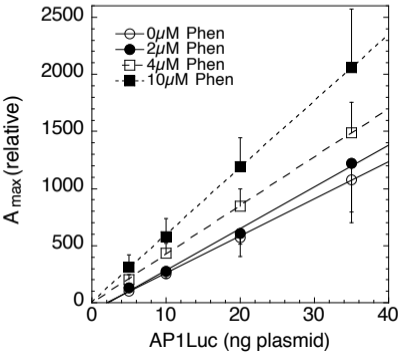

B

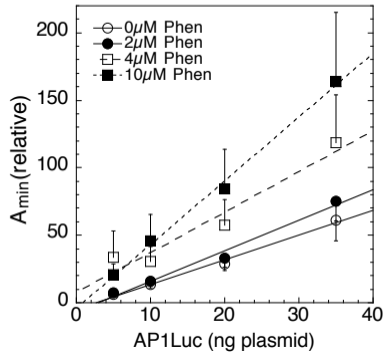

C

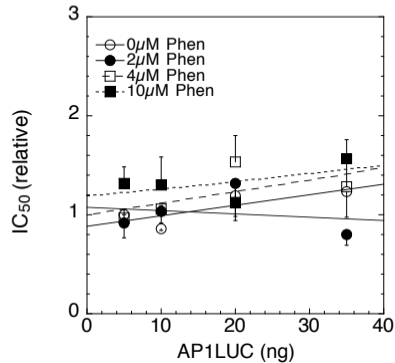

Supplement: S2 Fig — Experimental assays were conducted as in Fig. 5 with 10ng/ml of PMA and four concentrations of Dex. Average plots of (A) Amax, (B) Amin, and (C) IC50 vs. AP1LUC were obtained by first normalizing the data to the value for the lowest amount of AP1LUC and factor and then averaging and plotting the values (n = 5, ± S.E.M.). (PDF) [file pcbi.1004122.s002.pdf]
