# Supplementary material for: Kinetically-Defined Component Actions in Gene Repression
Source: PLoS Comput Biol. 2015 Mar 27;11(3):e1004122. doi: 10.1371/journal.pcbi.1004122 (PMC4376387; doi:10.1371/journal.pcbi.1004122)
Supplement: S1 Table — ε is a constant that is either 0 or 1. B parameters are positive constants that can take different values depending on context. These equations are derived in Dougherty et al. [22]. (DOCX) [file pcbi.1004122.s003.docx]

Table S1: Gene induction components in equation (2) where $X_{i}^{T}$ means total accelerator concentration at some step *i*:, $[D_{i}]$ means decelerator concentration at some step *i*:, $\alpha=0$ for *competitive* inhibition, $\gamma=0$ for *uncompetitive* inhibition, $\alpha=\gamma$ for *noncompetitive* inhibition, $q$ and $q'$are equilibrium or affinity constants. $\varepsilon$is a constant that is either 0 or 1. *B* parameters are positive constants that can take different values depending on context. These equations are derived in Dougherty et al. 2012 [22].

| 1. k < l < CLS |
| --- |
| 1. k < l = CLS |
| 1. k < CLS < l |
| 4. k = CLS < l |
| 5. CLS < k< l |
