# Supplementary material for: Kinetically-Defined Component Actions in Gene Repression
Source: PLoS Comput Biol. 2015 Mar 27;11(3):e1004122. doi: 10.1371/journal.pcbi.1004122 (PMC4376387; doi:10.1371/journal.pcbi.1004122)
Supplement: S2 Table — GR acts at location g and the factor acts at location k. GR as a repressor can act as either a decelerator anywhere or an accelerator after the CLS. The activated GR depends on steroid as [GR*] = G[S]/(K+[S]). Other parameters have the same meaning as in S1 Table. B parameters can have different values depending on context. The dose-response parameters are given by A max = T(0)/U(0), A min = T′/V′, IC 50 = T(0)/T′, and A max IC 50/A min = T(0)T′ (DOCX) [file pcbi.1004122.s004.docx]

Table S2: Dose-response parameter components for various cases of GR and a factor. GR acts at location *g* and the factor acts at location *k*. GR as a repressor can act as either a decelerator anywhere or an accelerator after the CLS. The activated GR depends on steroid as $[GR*]=G[S]/\left(K+[S]\right)$. Other parameters have the same meaning as in Table S1. *B* parameters can have different values depending on context. The dose-response parameters are given by$A_{\max}=T(0)/U(0)$, $A_{\min}=T'/V'$, $IC_{50}=T(0)/T'$, and $A_{\max}IC_{50}/A_{\min}=T(0)/T'$.

| 1. GR = *D*, *k < g* < CLS $T(0)=B_{2}q_{k}X_{k}^{T}(1+\alpha_{k}\beta_{k}q'_{k}[D_{k}])K$ |
| --- |
| 2. GR = *D*, g = k < CLS $T(0)=B_{2}q_{g}X_{g}^{T}K$ $U(0)=(1+B_{3})K+B_{4}q_{g}\varepsilon_{g}K+B_{5}q_{g}X_{g}^{T}K$ |
| 3. GR = *D*, *g* < *k* < CLS $T(0)=B_{2}q_{k}X_{k}^{T}(1+\alpha_{k}\beta_{k}q'_{k}[D_{k}])K$ |
| 4. GR = *D*, *g* < *k* = CLS $T(0)=B_{2}q_{k}X_{k}^{T}(1+\alpha_{k}\beta_{k}q'_{k}[D_{k}])K$ |
| 5. GR = *D*, *g* < CLS < *k* |
| 6. GR = *D*, *k* < *g =CLS* $T(0)=B_{2}q_{k}X_{k}^{T}(1+\alpha_{k}\beta_{k}q'_{k}[D_{k}])K$ |
| 7. GR = *D*, *k* = *g* = CLS $T(0)=B_{1}q_{g}X_{g}^{T}K$ $U(0)=(1+B_{3})K+B_{4}q_{g}K$ |
| 8. GR = *D*, *g* = CLS < *k* |
| 9. GR = *D*, *k* < CLS < *g* $T(0)=B_{1}q_{k}X_{k}^{T}(1+\alpha_{k}\beta_{k}q'_{k}[D_{k}])K$ |
| 10. GR = *D*, *k* = CLS *< g* |
| 11. GR = *D*, CLS< *k* <*g* |
| 12. GR = *D*, CLS < *g* = *k* $T(0)=B_{0}K+B_{1}q_{g}X_{g}^{T}K$ $U(0)=(1+B_{3})K+B_{4}q_{g}X_{g}^{T}K$ |
| 13. GR = *D*, CLS < *g* < *k* |
| 14. GR = A, *k* < CLS < *g* $T(0)=B_{1}q_{k}X_{k}^{T}(1+\alpha_{k}\beta_{k}q'_{k}[D_{k}])K$ |
| 15. GR = A, CLS = *k* < *g* $T(0)=B_{1}q_{k}X_{k}^{T}(1+\alpha_{k}\beta_{k}q'_{k}[D_{k}])K$ |
| 16. GR = A, CLS < *k* < *g* |
| 17. GR = A, CLS < *g* = *k* |
| 18. GR = A, CLS < *g* < *k* $T(0)=B_{0}K$ $U(0)=(1+B_{3})K$ |
