# Supplementary material for: Kinetically-Defined Component Actions in Gene Repression
Source: PLoS Comput Biol. 2015 Mar 27;11(3):e1004122. doi: 10.1371/journal.pcbi.1004122 (PMC4376387; doi:10.1371/journal.pcbi.1004122)
Supplement: S3 Table — (DOCX) [file pcbi.1004122.s005.docx]

Table S3: MCMC model fit results for TIF2

| Parameter | Predicted | | Permuted | | Unrestricted | |
| --- | --- | --- | --- | --- | --- | --- |
|  | ML | Mean (SD) | ML | Mean (SD) | ML | Mean (SD) |
| 1 | 4.6 | 9.6 (4.2) | 43 | 48 (11) | 51 | 60 (28) |
| 2 | 6.0 | 15 (8.6) | 0.58 | 1.4 (0.84) | 1.8 | 82 (50) |
| 3 | 2.4 | 4.3 (1.6) | 1.3 | 3.6 (2.5) | 3.9 | 7.7 (5.2) |
| 4 | 1.6 | 2.7 (0.86) | 94000 | 1700000 (650000) | 8.3 | 140 (84) |
| 5 | 27 | 30 (4.7) | 44000 | 740000 (370000) | 2000 | 32000 (9200) |
| 6 | 1.2 | 1.08 (0.09) | 1.4 | 1.5 (0.17) | 1.3 | 32 (23) |
| 7 | 0.26 | 0.20 (0.05) | 0.37 | 0.34 (0.035) | 1.2 | 1.9 (0.85) |
| 8 | 1.1 | 1.01 (0.093) | 0.00082 | 0.00011 (0.00026) | 3.0 | 28 (15) |
| 9 | - | - | - | - | 790 | 13000 (4800) |
| 10 | - | - | - | - | 1.2 | 0.69 (0.35) |
| 11 | - | - | - | - | 0.29 | 0.26 (0.050) |
| 12 | - | - | - | - | 0.96 | 0.45 (0.22) |

| Model | Predicted | | Permuted | | | Unrestricted | |
| --- | --- | --- | --- | --- | --- | --- | --- |
|  | ML | Mean | | ML | Mean | ML | Mean |
| Chi | 27.1 | 34.0 | | 83.2 | 89.4 | 25.9 | 38.1 |
| BIC | 60.4 | 67.3 | | 117 | 123 | 76.0 | 88.0 |
