# Supplementary material for: Kinetically-Defined Component Actions in Gene Repression
Source: PLoS Comput Biol. 2015 Mar 27;11(3):e1004122. doi: 10.1371/journal.pcbi.1004122 (PMC4376387; doi:10.1371/journal.pcbi.1004122)
Supplement: S4 Table — (DOCX) [file pcbi.1004122.s006.docx]

Table S4: MCMC model fits for NU6027

| Parameter | Predicted | | Permuted | | Unrestricted | |
| --- | --- | --- | --- | --- | --- | --- |
|  | ML | Mean (SD) | ML | Mean (SD) | ML | Mean (SD) |
| 1 | 26 | 28 (3.4) | 24 | 24 (2.3) | 24 | 27 (3.5) |
| 2 | 110000 | 66000 (38000) | 22 | 23 (2.4) | 18 | 19 (2.6) |
| 3 | 8.6 | 8.9 (0.85) | 3800 | 14000 (5400) | 240 | 1100 (650) |
| 4 | 36 | 42 (8.2) | 53000 | 310000 (210000) | 11000 | 59000 (58000) |
| 5 | 84000 | 49000 (28000) | 46000 | 260000 (170000) | 100000 | 450000 (250000) |
| 6 | 4.1 | 4.5 (0.60) | 183 | 670 (260) | 340000 | 2700000 (1100000) |
| 7 | 0.00033 | 0.00103(0.00098) | 0.0062 | 0.0021 (0.0011) | 29 | 35 (9.9) |
| 8 | 0.33 | 0.33 (0.030) | 0.00043 | 0.00015 (0.00014) | 20 | 22 (3.7) |
| 9 | - | - | - | - | 87000 | 350000 (190000) |
| 10 | - | - | - | - | 19000 | 140000 (58000) |
| 11 | - | - | - | - | 0.12 | 0.065 (0.074) |
| 12 | - | - | - | - | 0.0019 | 0.024 (0.0032) |

| Model | Predicted | | Permuted | | | Unrestricted | |
| --- | --- | --- | --- | --- | --- | --- | --- |
|  | ML | Mean | | ML | Mean | ML | Mean |
| Chi | 33.3 | 37.5 | | 34.3 | 37.9 | 28.7 | 34.4 |
| BIC | 66.5 | 70.8 | | 67.6 | 71.2 | 78.6 | 84.3 |
