# Supplementary material for: Kinetically-Defined Component Actions in Gene Repression
Source: PLoS Comput Biol. 2015 Mar 27;11(3):e1004122. doi: 10.1371/journal.pcbi.1004122 (PMC4376387; doi:10.1371/journal.pcbi.1004122)
Supplement: S5 Table — (DOCX) [file pcbi.1004122.s007.docx]

Table S5: MCMC model fit results for Phenanthroline

| Parameter | Predicted | | Permuted | | Unrestricted | |
| --- | --- | --- | --- | --- | --- | --- |
|  | ML | Mean (SD) | ML | Mean (SD) | ML | Mean (SD) |
| 1 | 3.6 | 4.0 (0.91) | 18 | 100 (130) | 5.0 | 5.5 (1.8) |
| 2 | 23 | 32 (18) | 730 | 3800 (2300) | 4.4 | 140 (110) |
| 3 | 4.0 | 4.7 (1.5) | 24 | 28 (11) | 21 | 32 (17) |
| 4 | 250000 | 180000 (68000) | 1200 | 8000 (6600) | 230 | 1700 (2100) |
| 5 | 130 | 160 (65) | 1500 | 3400 (3000) | 15000 | 1900000 (1100000) |
| 6 | 78000 | 49000 (17000) | 0.055 | 0.022 (0.028) | 1500000 | 50000000 (35000000) |
| 7 | 9800 | 5700 (2600) | 27 | 130 (84) | 1300000 | 60000000 (25000000) |
| 8 | 1.1 | 1.1 (0.16) | 0.017 | 0.016 (0.016) | 6300 | 1100000 (320000) |
| 9 | - | - | - | - | 59000 | 7300000 (4200000) |
| 10 | - | - | - | - | 460000 | 2400000 (3200000) |
| 11 | - | - | - | - | 58000 | 1900000 (970000) |
| 12 | - | - | - | - | 28 | 1500 (1000) |

| Model | Predicted | | Permuted | | | Unrestricted | |
| --- | --- | --- | --- | --- | --- | --- | --- |
|  | ML | Mean | | ML | Mean | ML | Mean |
| Chi | 23.8 | 30.0 | | 60.5 | 65.4 | 23.0 | 37.5 |
| BIC | 57.1 | 63.3 | | 93.8 | 98.6 | 72.9 | 87.4 |
