# Supplementary material for: Kinetically-Defined Component Actions in Gene Repression
Source: PLoS Comput Biol. 2015 Mar 27;11(3):e1004122. doi: 10.1371/journal.pcbi.1004122 (PMC4376387; doi:10.1371/journal.pcbi.1004122)
Supplement: S1 Text — (DOC) [file pcbi.1004122.s009.doc]

**Kinetically-defined component actions in gene repression**

**Supporting Information**

Carson C. Chow, Kelsey K. Finn, Geoffery B. Storchan, Xinping Lu, Xiaoyan Sheng, and S. Stoney Simons, Jr.

*Steps involved in running competition assays for gene repression*:

1) Seed U2OS.rGR cells in 24 well plates at 30,000 cells per well in DMEM. The 4x4 competition assay involves triplicates of four different steroid concentrations with all 16 combinations of four concentrations of two competitors for 192 wells (= 3x4x4x4).

2) Use X-tremeGENE HP (Roche; 0.8 µM/well) in OPTIMEM to transiently transfect triplicate wells of cells with plasmids for, in this instance, one of four different concentrations of AP1LUC reporter in four sets of 4x12 wells. Each well also contains Renilla TS plasmid (10 ng) as a control and the total DNA is adjusted to 300ng/well with pBSK^+^ DNA. When competing with a protein, each of the 4 sets of 12 wells containing one concentration of AP1LUC are cotransfected with 4 different concentrations of the plasmid for the protein with the molar amount of vector plasmid being kept constant by adding empty vector plasmid.

3) After 4h, change medium to DMEM plus 10% fetal bovine serum. After 20 h, add four appropriately spaced dilutions of steroid (in this case, Dex, which usually consists of an EtOH control and three Dex concentrations of 1x, 5x, and 25x) to all 16 sets of 12 wells in step 2 (one Dex concentration to three wells) in DMEM plus 10% fetal bovine serum. When a chemical is the competitor, one of four different concentrations of chemical in EtOH or DMSO (exact concentrations depend on the chemical but should be spaced to give adequate changes in total activity while preserving reasonable fold increase over EtOH) is also added to each sub-set of 12 in the set of 4x12 wells of step 2. The net result is that 4-point dose-response curves will be run on each of the 16 sets of 12 wells that contain all possible combinations of AP1LUC and chemical or protein cofactor. The final concentration of EtOH and DMSO in the media added to the cells is determined by toxicity studies with the cells being used. For U2OS.rGR cells, the final EtOH concentration is typically 0.1% but can be as high as 1%. The final concentration of DMSO should be ≤0.25%.

4) After 20h, lyse cells and determine luciferase and Renilla activities in cell lysates as recommended by Promega.

5) Normalize all Luciferase values for the level of Renilla in the same well to obtain values of Luciferase/Renilla.

6) Average each triplicate of Luciferase/Renilla and use these values to calculate “fold repression - 1” as ([(average activity with PMA plus EtOH)-(average activity with PMA and each Dex concentration)]/(average activity with PMA and highest Dex concentration)).

7) Plot “fold repression” of step 6 (= y) vs. Dex concentration (= x) and determine the best fit to a first-order Hill plot of y = m1*x/(m2 + x) to obtain maximal fold repression -1 (= m1) and IC_50_ (= m2). A_min_ is then calculated as A_max_/Fold repression, where Amax is the average Luciferase/Renilla value with EtOH in each set.

8) Calculate values for IC_50_, A_max_, A_min_, and A_max_*IC_50_/A_min_ for each of the 16 combinations. Normalize all of the values for IC_50_, A_max_, etc. in each experiment to one condition (e.g., EtOH with 5 ng AP1LUC and no competitor) and obtain averages for n independent experiments.

9) Use average, normalized values of step 8 to make graphs of IC_50_, A_max_, A_min_, and A_max_xIC_50_/A_min_ vs. both competitors, i.e., (a) vs. AP1LUC with the four different concentrations of chemical and (b) vs. chemical with the four different concentrations of AP1LUC.

10) Refer to Table 1 (see text) for correlation of characteristics of graphs of IC_50_, A_max_, A_min_, and A_max_xIC_50_/A_min_ from step 9 with the mechanistic explanation of how and where each of the two competing factors (in this case, AP1LUC and chemical) plus GR act when giving that particular graph. Table 1 is used to determine the actions of factor F1 first and then of factor F2.
